# Supplementary material for: The histone H4 lysine 20 demethylase DPY-21 regulates the dynamics of condensin DC binding
Source: J Cell Sci. 2022 Jan 26;135(2):jcs258818. doi: 10.1242/jcs.258818 (PMC8917352; doi:10.1242/jcs.258818)
Supplement: Supplementary information [file joces-135-258818-s1.pdf]

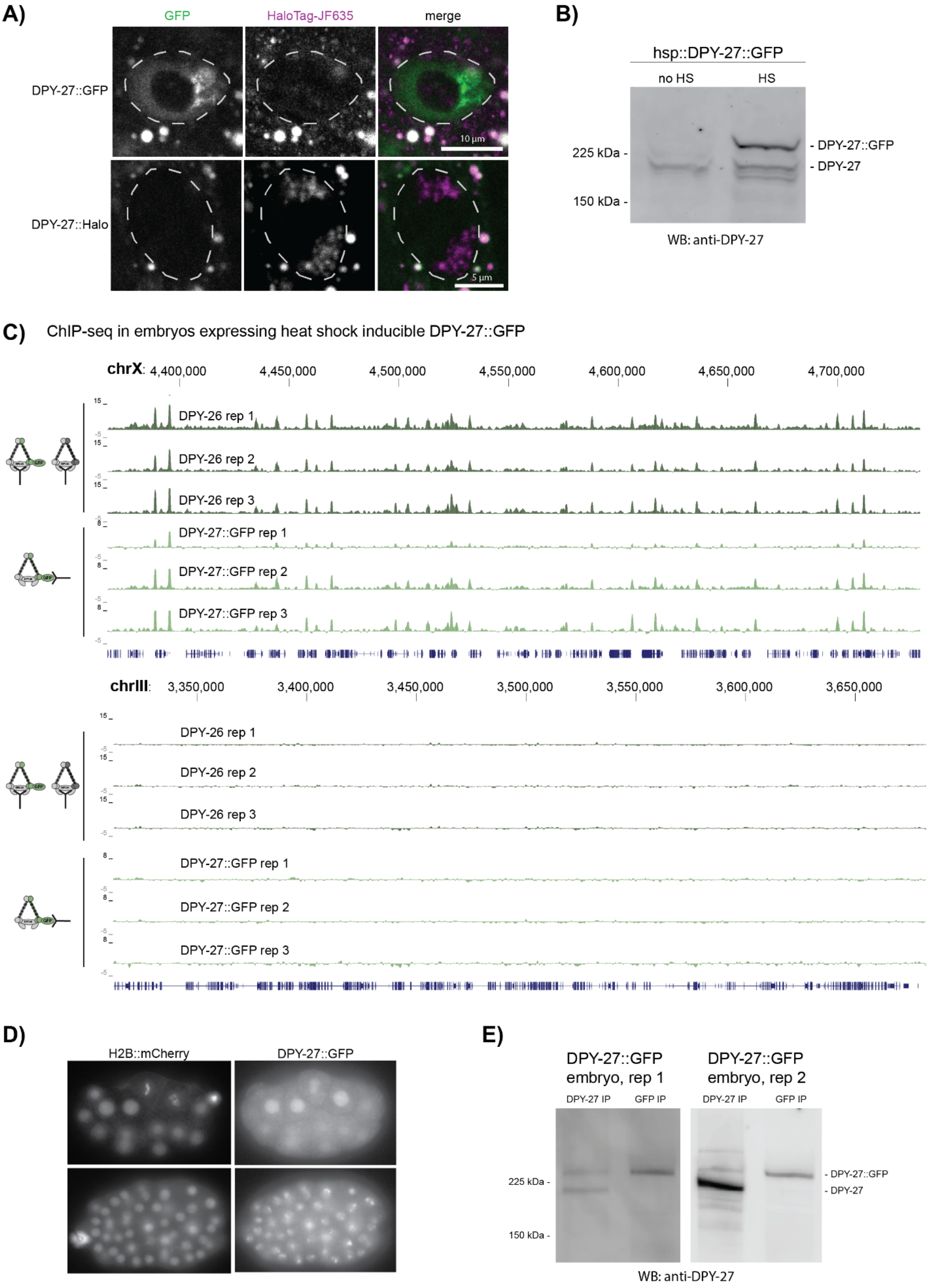

Journal of Cell Science • Supplementary information

Fig. S1. A) Validation of separable GFP and JF635-Halo signal. Fluorescent images of intestine nuclei after feeding JF635-Halo ligand in homozygous worms expressing heat-shock inducible DPY-27::GFP (upper row) or endogenously Halo-tagged DPY-27 (lower row). Both worm lines were stained with JF635-Halo ligand and heat-shocked. White dotted lines mark nuclei.

B) Protein extracts prepared from heat-shocked (HS) and non-heat-shocked (NHS) young adults carrying the *hsp::dpy-27::gfp* transgene were used for western blot. Incubation with DPY-27 antibody shows specific DPY-27::GFP expression upon heat shock.

C) Validation of DPY-27::GFP localization specifically to the X chromosomes by ChIP-seq. DPY-27::GFP ChIP-seq analysis replicates using an anti-GFP antibody in embryos. DPY-26 ChIP-seq was used as a positive control in the same extracts.

D) X-localization of DPY-27::GFP in embryos is indicated by subnuclear puncta that appears later in embryogenesis when condensin DC localizes specifically to the X chromosomes. H2B::mCherry and DPY-27::GFP signal 6 hours after a heat shock in early (before X localization and late embryos (after DC localization to the X).

E) Heat shock expression of DPY-27::GFP was variable in embryos. Two examples are shown where immunoprecipitation of DPY-27 showed different proportions of GFP tagged DPY-27 (top band) compared to endogenous (bottom band). Protein extracts were prepared from embryos isolated from gravid adults that were heat-shocked for 30 min at 35°C and recovered at room temperature for 2 hours.

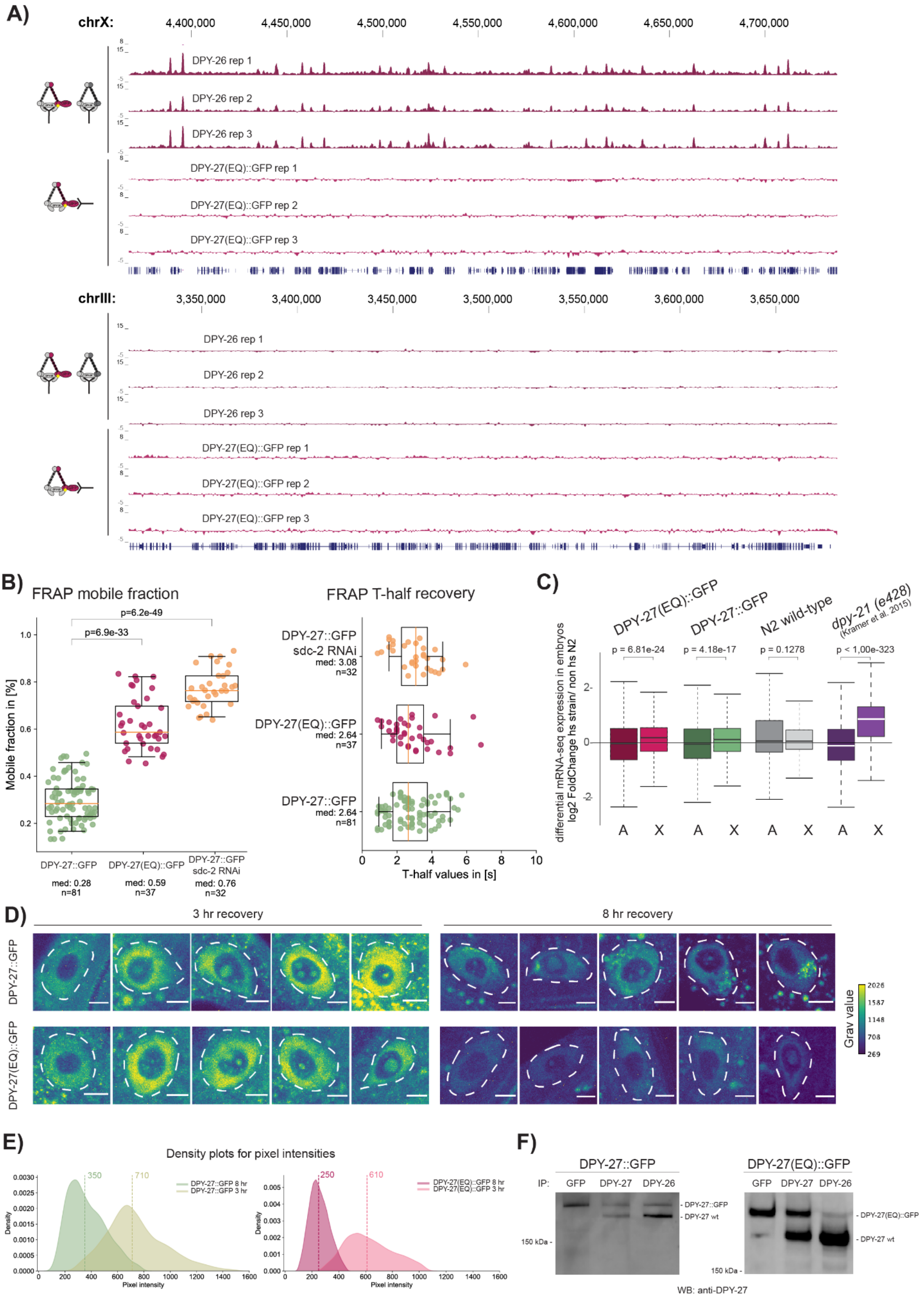

Journal of Cell Science • Supplementary information

Fig. S2. A) ChIP-seq data from replicates corresponding to Figure 2C. Replicates for the wild-type DPY-27::GFP ChIP-seq data can be found in Figure S1C.

B) FRAP analysis of mobile fractions (left panel) and T-half recovery time (right panel) corresponding to Figure 2D. P values are from an independent two-sample t-test.

C) Log2 fold changes in mRNA-seq between heat-shocked strains and non-heat-shocked wild-type embryos, collected after 30 min heat shock at 35°C followed by 2-hour recovery. Additionally, mRNA-seq log2 fold changes of non-heat-shocked *dpy-21 (e428)* from (Kramer et al., 2015). P values are from a Wilcoxon–Mann–Whitney test.

D) Example images of heat-shock expression of wild-type DPY-27::GFP and the ATPase mutant DPY-27(EQ)::GFP after 3 and 8 hours of recovery that were quantified in Supplemental Figure 2E. Images are normalized to the same gray values, and the scale bar corresponds to 5 µm.

E) Quantification of the GFP signal's pixel intensities in the nuclei after 3 and 8 hours of recovery from heat shock. The intensities were recorded from at least three biological replicates in adult intestine cells. For wild-type, DPY-27::GFP 21 images were used for the 3-hour intensity curve and 26 for the 8 hours recovery time point. For the intensity curves of DPY-27(EQ)::GFP, 44 images were used for the short time point and 36 images for the long recovery time point. Dotted lines indicate the median value for each distribution.

F) Co-immunoprecipitation analysis of condensin DC subunits in embryos. Protein extracts were prepared from embryos that were heat-shocked for 1 hour at 35°C and recovered at room temperature for 2 hours. Immunoprecipitated (IP) DPY-27::GFP and endogenous protein were analyzed by blotting with an anti-DPY-27 antibody. The intensity of the DPY-27::GFP and endogenous protein bands indicate their abundance in each immunoprecipitation.

Journal of Cell Science • Supplementary information

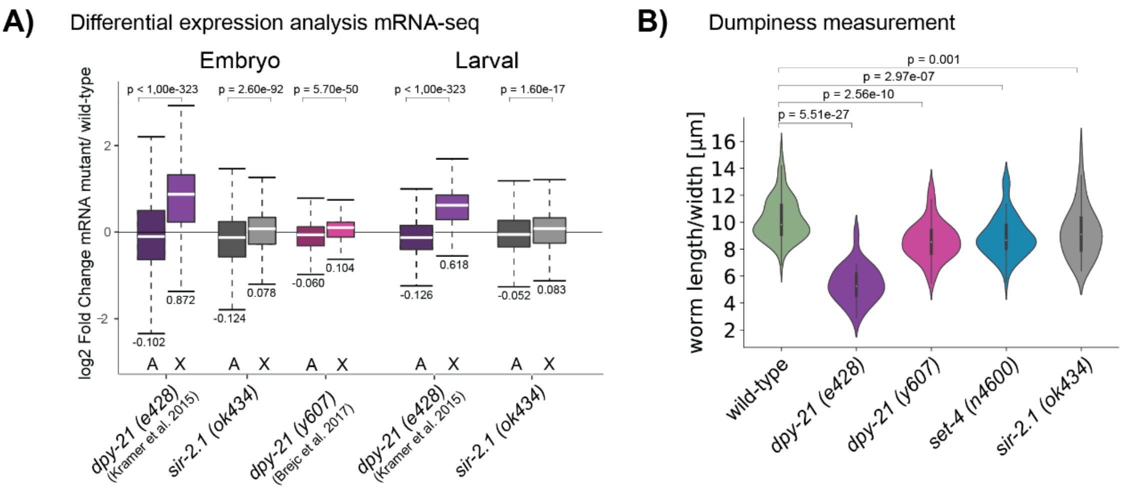

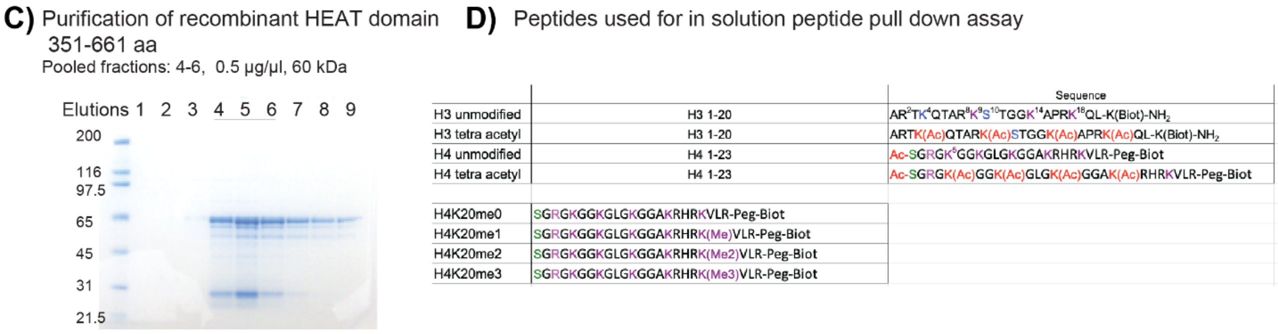

D) Peptides used for in solution peptide pull down assay

		Sequence
H3 unmodified	H3 1-20	AR^{2}TK^{4}QTAR^{8}K^{9}S^{10}TGGK^{14}APRK^{18}QL-K(Biot)-NH_2
H3 tetra acetyl	H3 1-20	ARTK(Ac)QTARK(Ac)STGGK(Ac)APRK(Ac)QL-K(Biot)-NH_2
H4 unmodified	H4 1-23	Ac-SGRGK^{5}GGKGLGKGGAKRHRKVLR-Peg-Biot
H4 tetra acetyl	H4 1-23	Ac-SGRGK(Ac)GGK(Ac)GLGK(Ac)GGAK(Ac)RHRKVLR-Peg-Biot
H4K20me0	SGRGKGGKGLGKGGAKRHRKVLR-Peg-Biot	
H4K20me1	SGRGKGGKGLGKGGAKRHRK(Me)VLR-Peg-Biot	
H4K20me2	SGRGKGGKGLGKGGAKRHRK(Me2)VLR-Peg-Biot	
H4K20me3	SGRGKGGKGLGKGGAKRHRK(Me3)VLR-Peg-Biot	

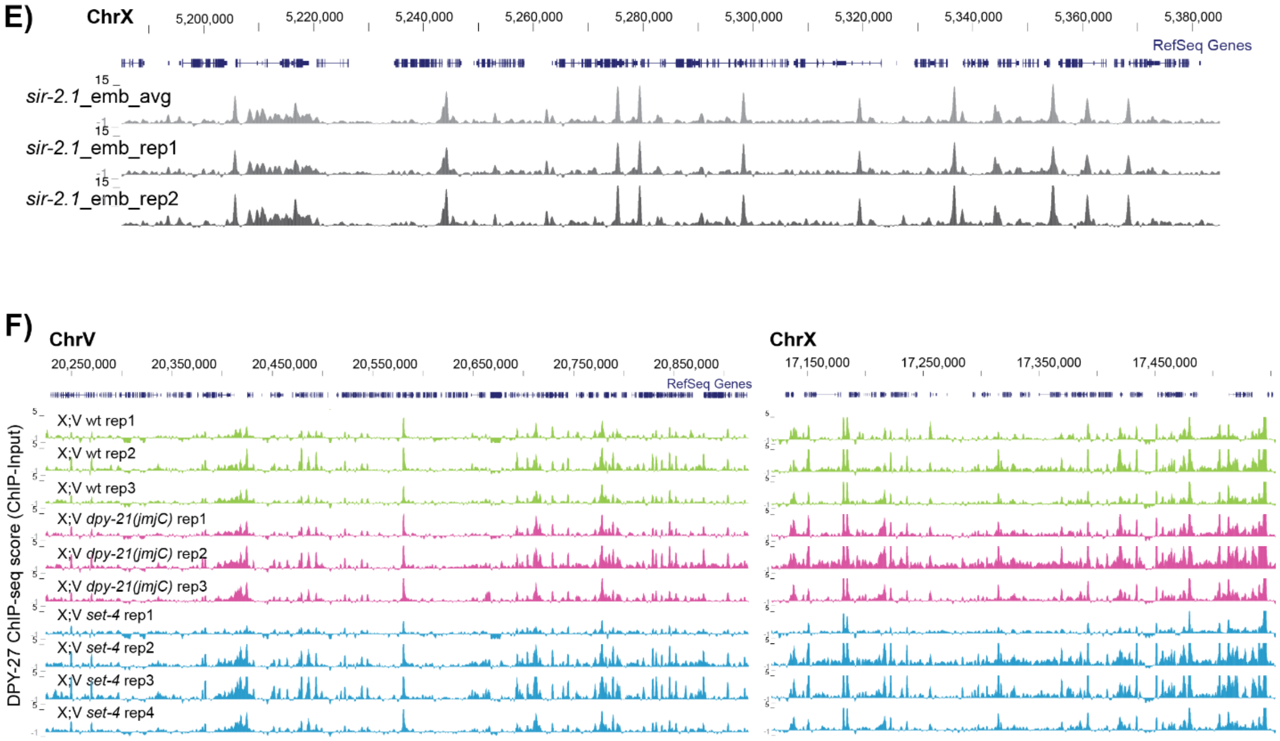

Journal of Cell Science • Supplementary information

Fig. S3. A) mRNA-seq analysis comparing published *dpy-21 (e428)* from (Kramer et al., 2015), *dpy-21(JmjC)* data from (Brejc et al., 2017), and new data in *sir-2.1* null mutant in embryos (left) and larvae (right). The level of X chromosome derepression compared to autosomes was compared in different mutants using log2 expression ratios compared to wild-type. Significant X chromosome upregulation was tested by a Wilcoxon–Mann–Whitney test. Median values of each group of genes are shown below each boxplot.

B) Dumpiness phenotype analysis of wild-type and different mutant worms. The length divided by the width of young adult worms was calculated as a proxy for their dumpiness level from two biological replicates. The following number of worms were used for each condition: wild-type: n= 102; *dpy-21(e428)*: n= 24; *dpy-21(y607)*: n= 67; *set-4 (n4600)*: n= 69; *sir-2.1(ok434)*: n= 51. P values are from an independent two-sample t-test.

C) Elutions of GST-DPY-28 HEAT repeat domain recombinant protein, predicted to be ~60 kDa. Fractions 4-6 were pooled for peptide binding assay.

D) Sequences and modifications of the N-terminal histone peptides analyzed.

E) UCSC genome browser shot of a representative region showing similar DPY-27 ChIP-seq patterns in *sir-2.1* replicates.

F) UCSC genome browser shot of replicates corresponding to Figure 3F. Genome browser view of DPY-27 ChIP-seq enrichment on the X chromosomal region of the X;V chromosome in X;V wild-type, *dpy-21(JmjC)* and *set-4* null backgrounds.

Journal of Cell Science • Supplementary information

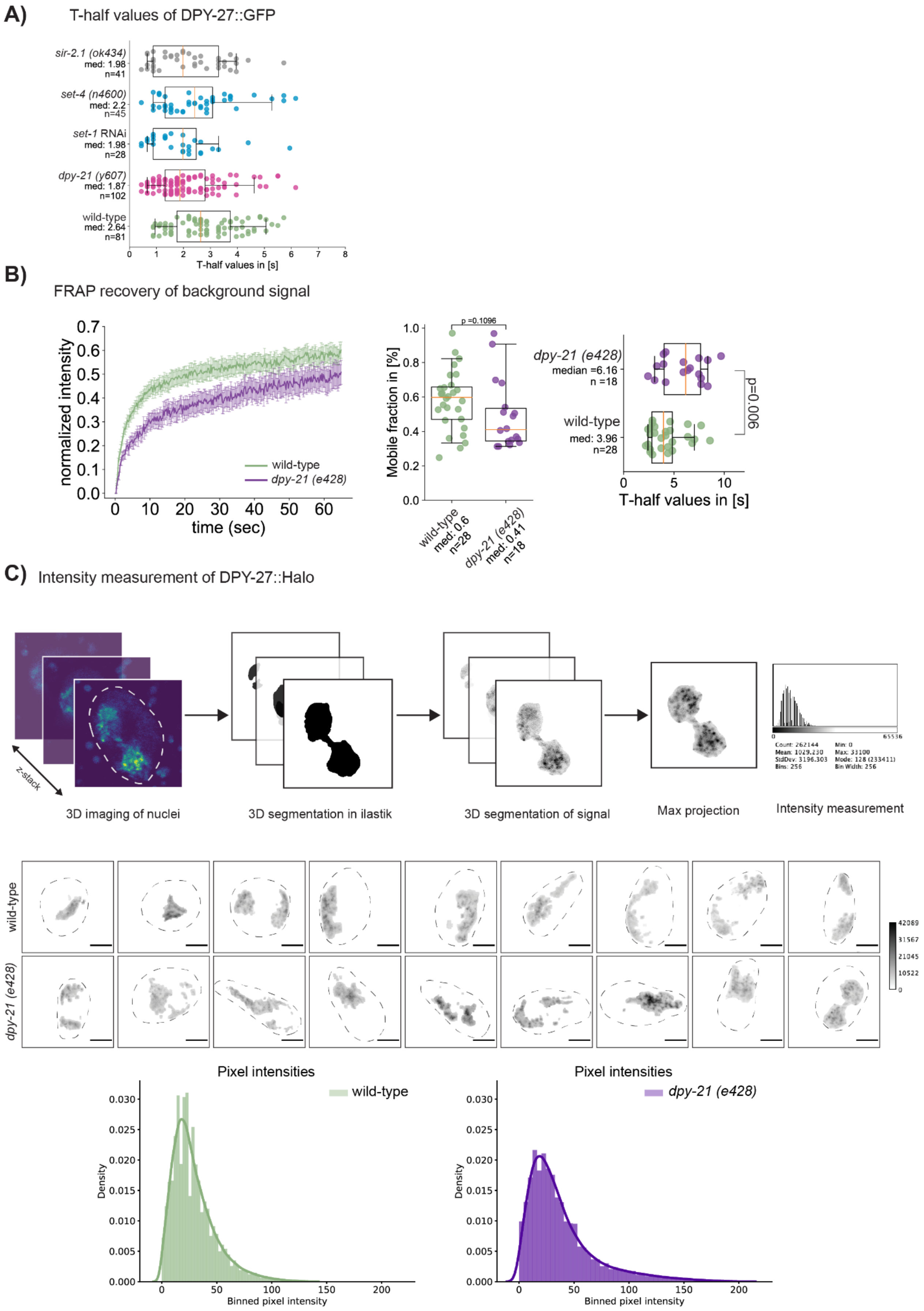
A)
T-half values of DPY-27::GFP
sir-2.1 (ok434) med: 1.98 n=41
set-4 (n4600) med: 2.2 n=45
set-1 RNAi med: 1.98 n=28
dpy-21 (y607) med: 1.87 n=102
wild-type med: 2.64 n=81
T-half values in [s]
B)
FRAP recovery of background signal
normalized intensity
time (sec)
wild-type
dpy-21 (e428)
p =0.1096
Mobile fraction in [%]
wild-type med: 0.6 n=28
dpy-21 (e428) med: 0.41 n=18
dpy-21 (e428) median =6.16 n =18
wild-type med: 3.96 n=28
p=0.006
T-half values in [s]
C)
Intensity measurement of DPY-27::Halo
z-stack
3D imaging of nuclei
3D segmentation in ilastik
3D segmentation of signal
Max projection
Intensity measurement
wild-type
dpy-21 (e428)
Pixel intensities
wild-type
Density
Binned pixel intensity
Pixel intensities
dpy-21 (e428)
Density
Binned pixel intensity

Fig. S4. A) T-half recovery time calculated from individual replicate FRAP recovery curves in Figure 4A.The T-half value for *dpy-21 (e428)* is not included in the plot due to the very low recovery during the experimental time frame.

B) Mean FRAP recovery curves for background DPY-27::GFP in wild-type and *dpy-21 (e428)* mutant worms. FRAPs were performed 8 hours after a 1-hour heat shock at 35°C. Unlike Figure 4A, the bleach point was not placed outside the X chromosomal area. Error bars for the bleach curves denote s.e.m. Number of bleached single intestine nuclei (from 2 biological replicates) for each experiment is n = 28 for wild-type and n = 18 for *dpy-21 (e428)*. The middle panel depicts the mobile fractions for the background recovery. The right panel depicts the T-half recovery times for the background recovery. P values are from an independent two-sample t-test.

C) Analysis of the fluorescence intensity of endogenously tagged DPY-27::Halo, for wild-type and *dpy-21 (e428)* worms corresponding to Figure 4C. The top row shows the analysis pipeline for the 3D segmentation of the HaloTag-JF549 signal. Z-stacks of intestine nuclei were imaged and segmented in 3D using ilastik (Berg et al., 2019). The resulting mask was used to segment the fluorescent signal in 3D, and from max projections, binned intensities were obtained. The middle row depicts example images of 3D segmented and max projected nuclei of the wild-type and mutant worms. The scale bar corresponds to 5 µm, and all images are calibrated to the same gray values. The bottom row shows the distribution of binned pixel intensities for wild-type and *dpy-21 (e428)* mutant worms from Figure 4C as two separate plots and with additional histograms underlying the density plot visualization from Figure 4C.

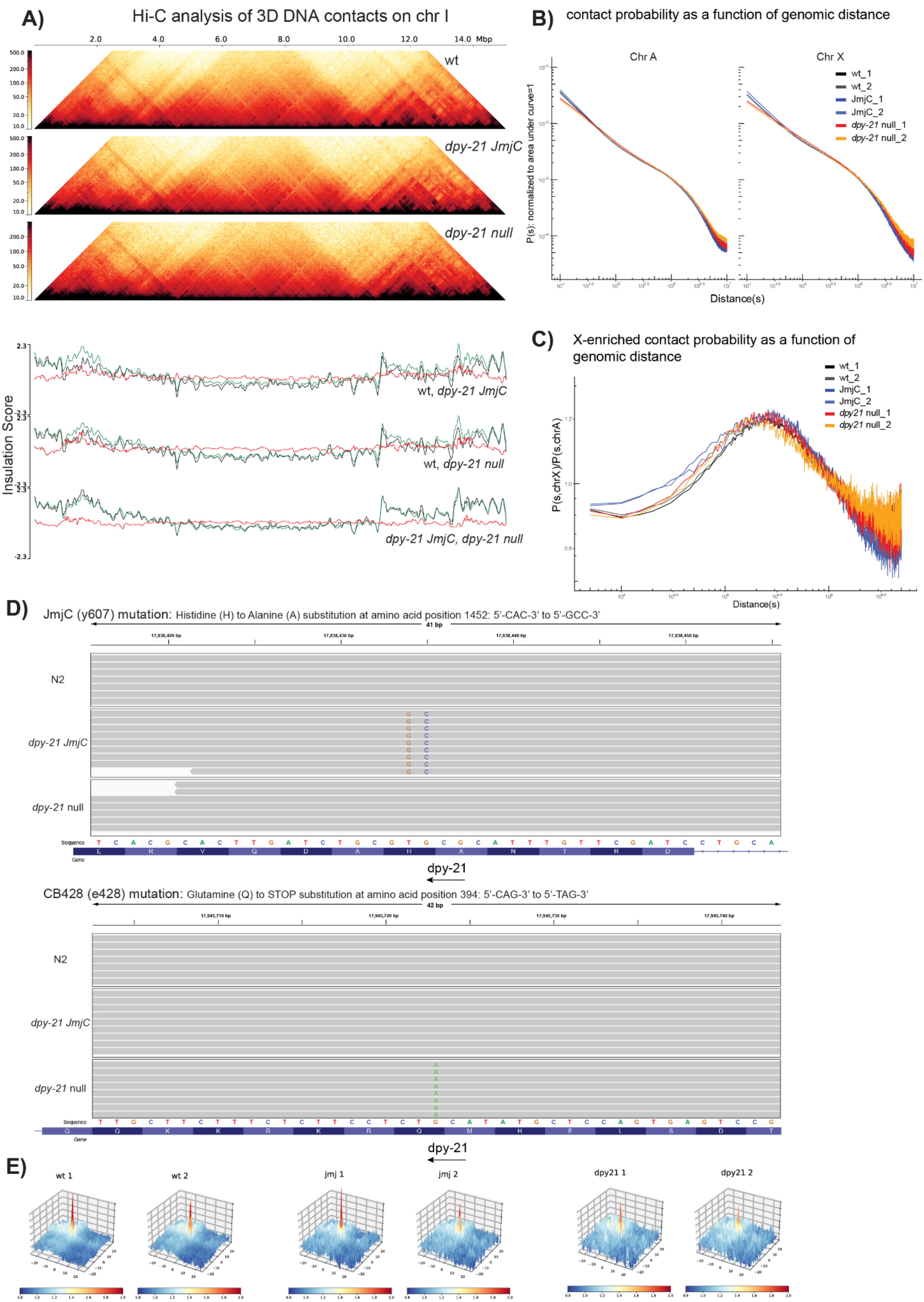

Journal of Cell Science • Supplementary information

Fig. S5. A) The same plot as Figure 5A for chromosome I.

B) Distance decay curve showing the relationship between 5- kb binned genomic separation, s, and average contact probability, P(s) for two biological replicates for each condition.

C) The same plot as Figure 5D for each replicate.

D) Hi-C reads were used to check for the validity of the strains. The reads were mapped to the ce10 genome. IGV snapshot of mapped reads sorted by mapping quality. Going top to bottom, the three samples correspond to the following genotypes: N2, *dpy-21 JmjC*, and *dpy-21 null*.

E) Meta-'dot' plot of individual biological replicates in wild-type, *dpy-21 JmjC (y607)* and *dpy-21* null mutant embryos from this study. Meta-'dot' plot showing the average strength of interactions between pairs of *rex* sites on a distance-normalized matrix. For 17 strong *rex* sites, a total of 33 *rex-rex* pairs located within 3 Mb of each other were used.

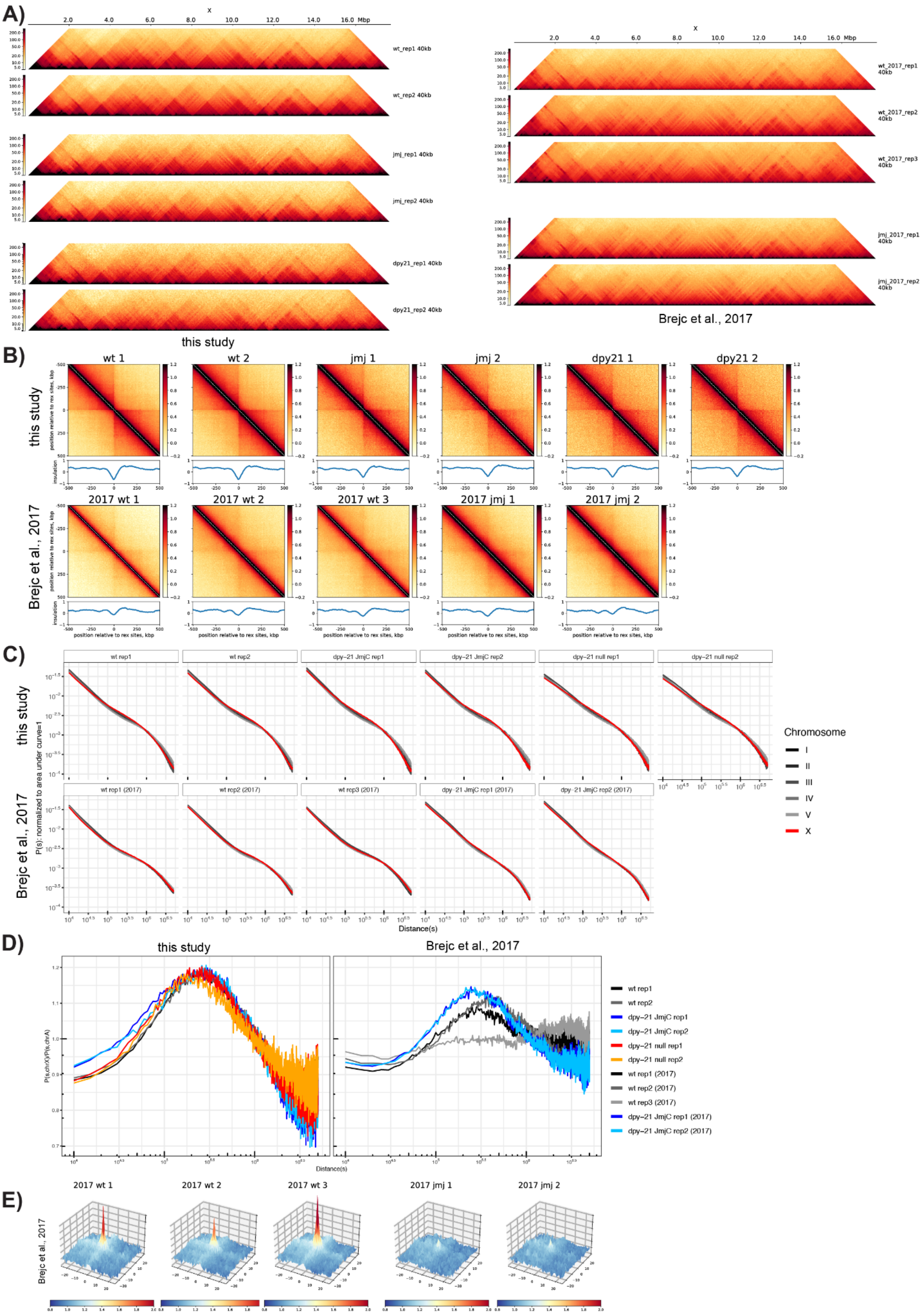

Journal of Cell Science • Supplementary information

Fig. S6. Hi-C analysis of individual biological replicates in wild-type, *dpy-21 JmjC (y607)*, and *dpy-21* null mutant embryos from this study and re-analysis of wild-type and *dpy-21 JmjC (y607)* from published data (Brejc et al., 2017).

A) Hi-C heatmap of chromosome-X showing wild-type, the *dpy-21 JmjC,* and the *dpy-21* null mutant from this study (left) and from the Brejc et al., 2017 study (right).

B) Pile-up analysis showing the average Hi-C map and the insulation scores +/- 500-kb surrounding the annotated 17 strong *rex* sites using data from this study (top row) and from the Brejc et al., 2017 study (bottom row).

C) Distance decay curve showing the relationship between 5-kb binned genomic separation, s, and average contact probability, P(s) computed per chromosome using data from this study (top row) and from the Brejc et al., 2017 study (bottom row).

D) X-enriched chromosomal contacts are visualized by an X/A normalized distance decay curve. For every genomic separation s, the unity normalized contact probability of X-chromosome, P(s,chrX), is divided by that of autosomes, P(s,chrA) from this study (left) and from the Brejc et al., 2017 study (right).

E) Meta-'dot' plot of individual biological replicates in wild-type and *dpy-21 JmjC (y607)* re-analysis of from published data (Brejc et al., 2017). Meta-'dot' plot showing the average strength of interactions between pairs of *rex* sites on a distance-normalized matrix. For 17 strong *rex* sites, a total of 33 *rex-rex* pairs located within 3 Mb of each other were used.

Journal of Cell Science • Supplementary information

Table S1. List of the C. elegans strains used in this study

strain name	***strain genotype***	***RRID of strain***	***short description***
N2	wild type	RRID:WB_STRAIN:N2	wild type laboratory strain
CB428	dpy-21(e428) V	RRID:WB_STRAIN:CB428	dpy-21 null
YPT47	X;V	RRID:WB_STRAIN:YPT47	X;V fusion in wild type background
MT14911	set-4 (n4600) II	RRID:WB_STRAIN:MT14911	set-4 null
ERC47	ersSi12[hsp16-41::dpy-27::GFP::3xFlag, unc-119(+)] II; unc-119(ed3) III		promoter_hsp::dpy-27::GFP in mossci Chr II site
ERC55	ersSi21[hsp16-41::dpy-27[EQ-TR]::GFP::3xFlag, unc-119(+)] II; unc-119(ed3) III		promoter_hsp::dpy-27 EQ TR mutation::GFP in mossci Chr II site
ERC57	set-4 (n4600) II; X;V (ypT47)		X;V fusion in set-4 null background
VC199	sir-2.1(ok434) IV	RRID:WB_STRAIN:VC199	sir-2.1 null
ERC76	ers53[dpy-27::halo] III		dpy-27::halo endogenous location fully complementing function with tag and 11 aa deletion
ERC81	dpy-21(y607)V; X;V (ypT47)		X;V fusion in dpy-21(*JmjC*) background (as in Brejc et al. 2017, introduced into X;V endogenously using CRISPR/Cas9)
TY5686	dpy-21(y607)		dpy-21*(JmjC*) catalytic mutant (Brejc et al. 2017)
RW10993	unc-119(ed3) III; itIs37 IV; stIs10116; wgIs94.		H2B-mCherry
strains derived for FRAP analysis by crossing			
SPL7	RW10993 X ERC55		
SPL8	RW10993 X ERC47		
SPL13	SPL8 X TY5686 (*dpy-21* y607 *JmjC* (codon 1454 changed to GCT from GAT))		
SPL14	SPL8 X MT14911 (*set-4* null)		
SPL15	SPL8 X CB428 (*dpy-21* null)		
SPL16	SPL8 X VC199		
SPL17	ERC47 X ERC76		
SPL18	RW10993 X ERC76		
SPL19	CB428 X ERC76		

Table S2. List of the primers used in this study

Target / purpose	F primer	R primer	Forward Sequence	Reverse Sequence	Product Size (bp)
amplify DPY-28 to be cloned into pGEX5X-2	DPY 28 351F	DPY-28 660R	atgcGGATCCGAACG AGCCGAAAAGCCA	atgcGAATTCTCAATC GTCCATTTGGGTTA G	
hsp promoter amplification from pCM1.57 with overlapping 15 bp to XhoI cut pCFJ151 for in fusion cloning, reverse primer is complementary to start of DPY-37 3' UTR		SE123R	GCAGGAATTCCTCG Actgcaggtcgactctaga	atatattacttcaatatttttctacc ggtacc	~550 bp
dpy-27 3'UTR amplification with overlap to pCJF151 on the right side	SE124F	SE124R	attgaagtaatatattttaaac	CACCGTACGTCTCG Attaggaaattatttttgat	~400 bp
amplify DPY-27 on the left overlapping with pCFJ151 with hsp promoter	SE135F	SE135R	gtattggtaccggtagaaaaaat ATGCAGCCGTTTAA AAGACG	ATGTCTGCTTCTTCG CACAC	~5.8 kb
amplify GFP3xflag on the left overlapping with DPY-27 and on the right with PCFJ151 dpy27 3' UTR	SE136F	SE136R	CGAAGAAGCAGAC ATATGAGTAAAGGA GAAGAACTT	gtttaaaatatattacttcaatTC ACTTGTCATCGTCAT CC	~1 kb
DPY-27 clone sequencing verification	SE127		gatgacgacaagggcagc		
DPY-27 clone sequencing verification	SE128		cgggcctgagatccacac		
DPY-27 clone sequencing verification	SE129		GCAAAGGATGAAG TTCGG		
DPY-27 clone sequencing verification	SE130		TGAACTGAAAGAG GCTGG		
DPY-27 clone sequencing verification	SE131		ATCACGCACTGGAA GCTC		

Journal of Cell Science • Supplementary information

DPY-27 clone sequencing verification	SE132		AATTGCAAACTTCA ACGg	
DPY-27 clone sequencing verification	SE133		AAGATGTTGACAAG TTCC	
DPY-27 clone sequencing verification	SE134		TCGAGGAGGAGATC AAAC	
Q5 mutagenesis of DPY-27 sequence for E-Q mutation	SE101F	SE101R	cAgATCGATGCGGC ACTGGAC	ATCCATCACGTAGA GGGGTG
crRNA from IDT to cut at 3' end of *dpy-27*	LS37		ACACGGCGTTGAA CGACAAT	
amplify halo from pLS19 with homology arms to tag DPY-27 C terminus. 5' SP9 modified primers	AM29F	AM29R	CATCTCCACCACCA ATCGTCGTCCAACG TCGCGTGCGAAGA AGCAGACATgggggag gaggatcgGAAATCGG TACAGGCTTTCC	ttttcaaaatttagtttaaaatatatt acttcaatTTATCCGGAG ATCTCGAGGGTGG
primers to detect halo insertion at 3' end of *dpy-27*	LS40F	LS40R	TGGACAGTACGTGA TGCAAAG	CGATGAGCCAGTAA GAAGACG
crRNA from IDT for JmjC dpy-21 H1452A	BR16_sg RNA		TTTCGACCTGAAAT TTCACG	
oligo repair template for JmjC dpy-21 mutant	BR16_oli go		TGGATCATCTTCAG TTGATTCACGCACT TGATCTGCGGCCGC ATTTGTTCGATCCTG CACAAAAATAGTTG AAATTTGAGTTTTT TGTAAATTTTAACA GTTTTTCAATAGAA AATTCGTATTCGTC GTGAAATTTCAGGT CGAAATGGGTTTTT TTTCGAAAACATTT GTGGTTGAAAAAGT GGCTCAGTCGGTAA GAT	
Amplify region of dpy-21 JmjC mutantion (product size 514bp. Not1 digestion products: 216bp - 298bp)	BR17F	BR17R	AACTATTGACCACA CCCGGG	GGCGGTTCGTAGAG ATCCAT

Journal of Cell Science • Supplementary information

Table S3. List of the antibodies used in this study

Target	Antibody	Antibody information	Antigen	RRID of antibody	Reference
DPY-27	JL00001	Rabbit polyclonal	1-409 aa	Covance Research Products Inc Cat# JL00001_DPY27, RRID:AB_2616039	Ercan et al 2007 Nature Genetics
DPY-26	JL00003	Rabbit polyclonal	740-1262 aa	Covance Research Products	Ercan et al 2009 Current Biology
MIX-1	JL00004	Rabbit polyclonal	837–1244 aa	Covance Research Products	Ercan et al 2009 Current Biology
GFP	ab290	Rabbit polyclonal	Recombinant full-length protein corresponding to GFP. Green fluorescent protein (GFP) from Aequorea victoria.	Abcam	--

Journal of Cell Science • Supplementary information

Table S4. Information for RNA-seq data used in this study

RNA-seq data from this study						
GEO accession number	***Sequencing ID***	***Description***	***Strain***	***Stage***	***Mapped reads***	***Technical Reps***
GSM5075626	SEA51	VC199_emb_rep1A	VC199	mixed embryos	13,342,597	SEA58 tech rep
GSM5075626	SEA58	VC199_emb_rep1B	VC199	mixed embryos	21,079,971	SEA51 tech rep
GSM5075627	SEA70	VC199_emb_rep2	VC199	mixed embryos	23,574,464	
GSM5075628	SEA77	VC199_emb_rep3	VC199	mixed embryos	17,555,806	
GSM5075629	SEA86	VC199_emb_rep4	VC199	mixed embryos	20,187,638	
GSM5075630	MK11	VC199_L2L3_Rep1A	VC199	L2-L3	15,784,655	MK25 tech rep
GSM5075630	MK27	VC199_L2L3_Rep1B	VC199	L2-L3	16,716,809	MK19 tech rep
GSM5075631	MK19	VC199_L2L3_Rep2	VC199	L2-L3	10,497,663	
GSM5075632	MK46	VC199_L2L3_Rep3	VC199	L2-L3	21,498,117	
GSM5075633	MK60	VC199_L2L3_Rep4	VC199	L2-L3	24,037,603	
GSM5075634	SEA224	KB01_emb_RNA_rep1	KB01	mixed embryos	8,220,567	
GSM5075635	SEA225	KB01_emb_RNA_rep2	KB01	mixed embryos	9,726,617	
GSM5075636	LAS41	KB01_emb_RNA_rep3	KB01	mixed embryos	24,873,910	
GSM5075637	SEA221	MK14_emb_RNA_rep1	MK14	mixed embryos	9,012,229	
GSM5075638	SEA222	MK14_emb_RNA_rep2	MK14	mixed embryos	10,363,195	
GSM5075639	SEA223	MK14_emb_RNA_rep3	MK14	mixed embryos	7,729,455	
GSM5075640	LAS47	LS_ext534_emb_RNA_N2_HS_rep1	N2, heat shock	mixed embryos	25,478,937	
GSM5075641	LAS48	LS_ext536_emb_RNA_N2_HS_rep2	N2, heat shock	mixed embryos	18,412,383	
GSM5075642	LAS49	LS_ext549_emb_RNA_N2_HS_rep3	N2, heat shock	mixed embryos	24,768,721	

Journal of Cell Science • Supplementary information

Published RNA-seq data used in this study

GEO accession number	***Strain***	***Description***	***Stage***	***Reference***		
GSE67650	N2	N2	mixed embryo	Kramer et al PLoS Gen 2015		
GSE67650	CB428	dpy-21 null	mixed embryo	Kramer et al PLoS Gen 2015		
GSE67650	N2	N2	L3	Kramer et al PLoS Gen 2015		
GSE67650	CB428	dpy-21 null	L3	Kramer et al PLoS Gen 2015		
GSE84581	N2	N2	mixed embryo	Brejc et al Cell 2017		
GSE84581	TY5686	dpy-21 (y607)	mixed embryo	Brejc et al Cell 2017		

Table S5. Sanger sequencing results for ERC76

Sequencing results for ERC76, Halo CRISPR tagging dpy-27, revealed insertion of unknown sequence (grey) before the tag sequence, which does not affect dpy-27 function					
ATTGATCGAAGAAGCAACTCCATCTCCACCACCAATCCACTCCTCGTCGAAGGTCGAAATCGGTAC AGGCTTTCCATTCGACCCCCATTATGTGGAGGTCCTCGGAGAGCGTATGCACTACGTCGACGTCGG ACCACGTGACGGAACCCCAGTCCTCTTCCTCCACGGAAACCCAACCTCCTCCTACGTCTGGCGTA ACATCATCCCACACGTCGCCCCAACCCACCGTTGCATCGCCCCAGACCTCATCGGAATGGGAAAGT CCGACAAGCCAGACCTCGGATACTTCTTCGACGACCACGTCCGTTTCATGGACGCCTTCATCGAGG CCCTCGGACTCGAGGAGGTCGTCCTCGTCATCCACGACTGGGGATCCGCCCTCGGATTCCACTGGG CCAAGCGTAACCCAGAGCGTGTCAAGGTAAGTTTAAACATATATATACTAACTAACCCTGATTATTT AAATTTTCAGGGAATCGCCTTCATGGAGTTCATCCGTCCAATCCCAACCTGGGACGAGTGGCCAGA GTTCGCCCGTGAGACCTTCCAAGCCTTCCGTACCACCGACGTCGGACGTAAGCTCATCATCGACCA AAACGTCTTCATCGAGGGAACCCTCCCAATGGGAGTCGTCCGTCCACTCACCGAGGTCGAGATGG ACCACTACCGTGAGCCATTCCTCAACCCAGTCGACCGTGAGCCACTCTGGCGTTTCCCAAACGAG CTCCCAATCGCCGGAGAGCCAGCCAACATCGTCGCCCTCGTCGAGGAGTACATGGACTGGCTCCA CCAATCCCCAGTCCCAAAGCTCCTCTTCTGGGGAACCCCAGGAGTCCTCATCCCACCAGCCGAGG CCGCCCGTCTCGCCAAGTCCCTCCCAAACTGCAAGGTAAGTTTAAACAGTTCGGTACTAACTAACC ATACATATTTAAATTTTCAGGCCGTCGACATCGGACCAGGACTCAACCTCCTCCAAGAGGACAACC CAGACCTCATCGGATCCGAGATCGCCCGTTGGCTCTCCACCCTCGAGATCTCCGGATAAattgaagtaata tattttaaactaaattttgaaaaaaaaaagaaaactttgtgaaaaatccaaaaaatgagaccaactttcttt					
dpy-27					
insertion					
halo					
stop codon					

Table S6. Sanger sequencing results for ERC81

Sequencing results showing CAC to GCC change to generate the X;V, dpy-21(JmjC y607)				
NNNNNNNNNNNNNNNNNNNNTGGTAGANGACCATCTTACCGACTGAGCCACTTTTTCAACCACA AATGTTTTCGAAAAAAAACCCATTTCGACCTGAAATTTCACGACGAATACGAATTTTCTATTGAAA AACTGTTAAAATTTACAAAAAACTCAAATTTCAACTATTTTTGTGCAGGATCGAACAAATGCGGCC GCAGATCAAGTGCGTGAATCAACTGAAGATGATCCAACAACAACAACAACAACTACAACGACTAC AAGTTCTTCTTCTTCTTCAAAATCGAAAAAATCGGCGAAATCCGATCCGACATTTGTTAAATCA ACGGCTGCTGTGGGTGTCCTACAGGGTATCAGGAATCCTGATGCAAATGACGATGATGAATATTATG AGGATGAACGAAAAGCTGTTAAAGAAGTTATTGTATTTGATGCACATGATTTGCATAAAGTTGCACA TCATCTTGCAATGGATCTCNNNAAANCCGCCA				
crRNA				
disrupted PAM site				
changed codon				

Table S7. Information for ChIP seq data used in this study

Click here to download Table S7

Table S8. Information for Hi-C data used in this study

Click here to download Table S8

Table S9. Readme for DEseq output

**Gene annotations are from WS220 (UCSC genome version ce10).**	
**Transcripts Per Kilobase Million (TPM) tab**	
TPM:	
sample_sums<-apply(data.matrix(fpkm[,-c(1:2)]),2,sum)	
tpm<-t(t(fpkm[,-c(1:2)])/sample_sums)*10^6	
**DEseqOutput Tabs**	
column name:	
gene	name of gene
wbid	wormbase ID of the gene
baseMean	average of normalized count values
log2FoldChange	log2 fold change effect size estimate
lfcSE	standard error estimate for the log2 fold change values
stat	Wald statistic
pvalue	Wald test p-value
padj	Benjamini-Hochberg adjust p-value

Journal of Cell Science • Supplementary information

Table S10. TPM replicates

Click here to download Table S10

Table S11. DEseq Output VC199/N2 embryo

Click here to download Table S11

Table S12. DEseq Output VC199/N2 L3

Click here to download Table S12

Table S13. DEseq Output MK14/N2 embryo HS

Click here to download Table S13

Table S14. DEseq Output KB01/N2 embryo HS

Click here to download Table S14

Journal of Cell Science • Supplementary information
