# Supplementary material for: The histone H4 lysine 20 demethylase DPY-21 regulates the dynamics of condensin DC binding
Source: J Cell Sci. 2022 Jan 26;135(2):jcs258818. doi: 10.1242/jcs.258818 (PMC8917352; doi:10.1242/jcs.258818)
Supplement: Reviewer comments [file joces-135-258818_review_history.pdf]

Journal of Cell Science

The histone H4 lysine 20 demethylase DPY-21 regulates the dynamics of condensin DC binding

Laura Breimann, Ana Karina Morao, Jun Kim, David Sebastian Jimenez, Nina Maryn, Krishna Bikkasani, Michael J. Carrozza, Sarah E. Albritton, Maxwell Kramer, Lena Annika Street, Kustrim Cerimi, Vic-Fabienne Schumann, Ella Bahry, Stephan Preibisch, Andrew Woehler and Sevinc Ercan
DOI: 10.1242/jcs.258818

Editor: Caroline Hill

Review timeline

Original submission:	21 April 2021
Editorial decision:	1 June 2021
First revision received:	29 September 2021
Editorial decision:	28 October 2021
Second revision received:	18 November 2021
Accepted:	29 November 2021

Original submission

First decision letter

MS ID#: JOCES/2021/258818

MS TITLE: A noncatalytic activity of the H4K20 demethylase DPY-21 regulates condensin DC binding

AUTHORS: Laura Breimann, Ana Karina Morao, Jun Kim, David Jimenez, Nina M. Maryn, Krishna Bikkasani, Michael J Carrozza, Sarah Elizabeth Albritton, Maxwell Kramer, Lena Annika Street, Kustrim Cerimi, Vic-Fabienne Schumann, Ella Bahry, Stephan Preibisch, Andrew Woehler, and Sevinc Ercan
ARTICLE TYPE: Research Article

We have now reached a decision on the above manuscript.

To see the reviewers' reports and a copy of this decision letter, please go to: https://submit-jcs.biologists.org and click on the 'Manuscripts with Decisions' queue in the Author Area. (Corresponding author only has access to reviews.)

As you will see, the reviewers raise a number of substantial criticisms that prevent me from accepting the paper at this stage. They suggest, however, that a revised version might prove acceptable, if you can address their concerns. If you think that you can deal satisfactorily with the criticisms on revision, I would be pleased to see a revised manuscript. We would then return it to the reviewers.

The main criticism from reviewers 2 and 3 is that the paper is not one coherent story. There is the first part focused on DPY-27 and the second part on the non-catalytic function of DPY-21. It will be important in the revisions to try to meld the two stories together better and to change the title to reflect all the findings. Reviewers 2 and 3 make suggestions of how this can be achieved. In addition, reviewer 1 points out that some conclusions in the manuscript are over-reliant on qualitative data that could be strengthened with quantification and statistical analysis. In other places the text contradicts or ignores figure data. It will be important to address both of these

© 2021. Published by The Company of Biologists under the terms of the Creative Commons Attribution License (https://creativecommons.org/licenses/by/4.0/).

issues in the revised manuscript. It will also be important to perform the controls for the IPs requested by reviewer 3.

We are aware that you may be experiencing disruption to the normal running of your lab that makes experimental revisions challenging. If it would be helpful, we encourage you to contact us to discuss your revision in greater detail. Please send us a point-by-point response indicating where you are able to address concerns raised (either experimentally or by changes to the text) and where you will not be able to do so within the normal timeframe of a revision. We will then provide further guidance. Please also note that we are happy to extend revision timeframes as necessary.

Please ensure that you clearly highlight all changes made in the revised manuscript. Please avoid using 'Tracked changes' in Word files as these are lost in PDF conversion.

I should be grateful if you would also provide a point-by-point response detailing how you have dealt with the points raised by the reviewers in the 'Response to Reviewers' box. Please attend to all of the reviewers' comments. If you do not agree with any of their criticisms or suggestions please explain clearly why this is so.

Reviewer 1

Advance summary and potential significance to field

Condensins play important roles in chromatin compaction, influencing cell division, nuclear organization and transcription. Breimann et al demonstrate the ability to monitor dynamics of the C elegans specific condensin DC complex, which performs dosage compensation in XX hermaphrodites. This analysis was done in young adult intestinal cells, which are postmitotic and polyploid. Chromatin association of condensin DC was dependent on ATP hydrolysis by the DC-specific SMC homolog DPY-27 and the complex recruiting SDC-2. Despite many histone post-translational modifications being associated with dosage compensation, several genes encoding DC-associated PTM writer proteins do not appear to affect condesin DC dynamics, with the exception of DC-associated DPY-21. However, its recently described H4K20 KDM activity was not responsible for its function in promoting condensin motility; rather, this enzymatic activity was described earlier as being necessary for normal contact range in cis on X.
This report provides a valuable survey of different attributes of the condensin DC complex that govern its behavior (ATP hydrolysis, domains of interaction with chromatin, dynamic association with chromatin), as well as its relationship with chromatin PTMs. In certain places outlined below however, the conclusions in the manuscript are over-reliant on qualitative data that can be strengthened with quantification and statistical analysis. In other places text contradicts or ignores figure data. Addressing these issues should involve straightforward analysis of existing data. If appropriately carried out, in my opinion this manuscript would be suitable for publication.

Comments for the author

Condensins play important roles in chromatin compaction, influencing cell division, nuclear organization and transcription. Breimann et al demonstrate the ability to monitor dynamics of the C elegans specific condensin DC complex, which performs dosage compensation in XX hermaphrodites. This analysis was done in young adult intestinal cells, which are postmitotic and polyploid. Chromatin association of condensin DC was dependent on ATP hydrolysis by the DC-specific SMC homolog DPY-27 and the complex recruiting SDC-2. Despite many histone post-translational modifications being associated with dosage compensation, several genes encoding DC-associated PTM writer proteins do not appear to affect condesin DC dynamics, with the exception of DC-associated DPY-21. However, its recently described H4K20 KDM activity was not responsible for its function in promoting condensin motility; rather, this enzymatic activity was described earlier as being necessary for normal contact range in cis on X.

This report provides a valuable survey of different attributes of the condensin DC complex that govern its behavior (ATP hydrolysis, domains of interaction with chromatin, dynamic

© 2021. Published by The Company of Biologists under the terms of the Creative Commons Attribution License (https://creativecommons.org/licenses/by/4.0/).

association with chromatin), as well as its relationship with chromatin PTMs. In certain places outlined below, however, the conclusions in the manuscript are over-reliant on qualitative data that can be strengthened with quantification and statistical analysis. In other places text contradicts or ignores figure data. Addressing these issues should involve straightforward analysis of existing data. If appropriately carried out, in my opinion this manuscript would be suitable for publication.

Main points

Fig 2E: The ratio of endogenous DPY-27 to DPY-27::GFP signal should be calculated and graphed in both WT and EQ contexts to support the authors' assertion that EQ is less likely to interact with DPY-26 than WT. The images show proportional decreases of both endogenous and tagged DPY-27 at both time points in WT and EQ strains. Furthermore, the authors claims regarding proteins stability (discussion of Fig 2B in text) seems out of sync with the data. "Directly after heat shock… expressed at similar levels" the figure does not show signal intensity directly after heat shock (what time point does 'directly' indicate?). Add pixel intensity of this 'direct' time point to the graphs. By 8 hr, both WT and EQ appear similar in their signal, also undermining claims of EQ-specific protein degradation.

The authors should provide median signal intensity for the 'direct after heat shock', 3hr and 8 hr time point to clarify these claims. As currently shown, there is less overall decrease of signal in the EQ mutant going from 3 hr to 8 hr (WT (3x less, EQ 2x less). The relevant section of the discussion should be modified accordingly. Also, the methods suggest that separate mosSCI transgenes, one WT and one EQ, were separately integrated. Independent mosSCI integration events, even of the same transgenes, can show quite variable expression. Therefore, any discussion of protein levels needs to explicitly acknowledge this caveat to interpreting total protein levels.

HEAT in vitro binding of recombinant histone peptides: The figures show data consistent with worm dosage compensation. However, either the text or the representative image does not clearly describe the K20 methylation data. Specifically, there appears to be no difference in K20me0 and K20me1, with less binding to me2/3. Yet, the text says 'increased methylation of lysine 20 [relative to unmodified, from previous sentence] reduced the interaction. The authors need to quantify the binding intensities, normalized to peptide loading, and display the ratios me1 vs me0, me2 vs me0, me3 vs me0, etc. They need to perform statistical analysis of the difference in signal, and indicate in the legend how many independent replicates were analyzed. Finally the text would need to be appropriately modified. Since DC is associated with K20me1 enrichment at the expense of K20me2/3, this difference matters.

The RNAi feeding strategy is missing from the methods. This should be added. Specifically, it is problematic for interpreting set-1 RNAi experiments (Fig 4A/B). If knockdown of set-1 is induced after embryogenesis, it is possible that its role in condensin DC motility may have already been performed. Therefore, set-1 RNAi feeding should be started no later than the late larval stage L4 in the generation prior to the one in which animals are imaged. The authors are requested to clarify the method used and, if necessary, address the timing of RNAi addition with new experiments.

SET-4 has been implicated in DC in several studies. Here, the authors show that loss of set-4 increases dumpiness in animals. In Fig 4A set-4 also seems to decrease FRAP recovery. However, the authors ignore this difference. Please clarify either with statistical measurements or supporting information that justifies this interpretation of the data. Furthermore, the authors' claim that the H4K16/K20 modifiers act downstream of condensin DC is incompletely explored. Specifically, perhaps H4K20me2 traps condensin DC on chromatin in the absence of DPY-21 protein, and the non-catalytic activity senses or reduces interaction with this PTM in some way. The authors are encouraged to perform FRAP recovery in animals null for both dpy-21 and set-4 alleles to address this question.

Missing is some comment regarding the nature of the non-catalytic activity. Is there structural analysis possible that would provide a testable basis for a mechanism (e.g. the

© 2021. Published by The Company of Biologists under the terms of the Creative Commons Attribution License (https://creativecommons.org/licenses/by/4.0/).

K20me2 experiment proposed above)?

Finally, the manuscript would benefit from performing FRAP experiments also in early embryos because comparisons could be made between condensin DC dynamics in dividing and non-dividing cells. Although HS induction may be variable, the images in Fig S1C suggest the localization to X is discernible, and C. elegans embryonic subnuclear concentration of proteins have been successfully FRAP'ed before. Imaging embryos between the 50-100 cell stage would likely yield nuclei sufficiently large to FRAP at a developmental stage in which DC has been activated.

Minor points

Even though the endogenously tagged GFP allele was not suitable for analysis, please include the details of insert preparation in the methods as done for the halo tag. NOTE: "Generation of Halo::DPY-27 strain" should be written DPY-27::Halo if, as the method says, Halo was attached to the C-terminus.

Fig 1B: indicate the post-heat shock time point.

Fig 1C: show representative images for 0/3/8 hr.

Fig 1D: indicate visually and in the legend that anti-DPY-27 antibody was used for blotting.

Fig 1G: indicate the time point at which the mobility fraction is calculated

"To test if the failure of DPY-27(EQ)::GFP to bind is due to its inability to form a complex, we performed co-immunoprecipitation experiments (Figure 2F)." Should be 2E.

Fig S2C Add X:A RNAseq data from a DC mutant to this graph to provide context. Using the dpy-21 (Kramer 2015) data again would suffice. Please also address in the text that there is X:A imbalance in the WT DPY-27::GFP strain.

"Thus, set-1, set-4, sir-2.1, and the catalytic activity of dpy-21 act downstream of condensin DC…" binding? Dynamics? Clarify which aspect of condensin these genes act downstream of, since they participate in X chromosome silencing too. The same comment is relevant to the discussion: "Thus, H4K20me1 and H4K16ac act downstream of condensin DC [what – binding or dynamics ?] to repress transcription."

Same problem in first two discussion paragraphs: "also eliminates DPY-27 binding (to what) (Fig 2)" and DPY-27 EQ mutation eliminating its binding (to what), measured by…"

Fig S3E WT tracks missing from figure but indicated as present in figure legend.

17 nuclei (Fig 4C) are insufficient to 1) get such a smooth a distribution and 2) to be robustly compared with 31 dpy-21 nuclei, to give a tail of high signal. The difference should be tested with statistics and more control nuclei should be scored..

<u>Reviewer 2</u>

Advance summary and potential significance to field

This manuscript by Breimann et al contains a somewhat interesting collection of observations related to the dosage compensation complex in C. elegans. Even though some of the results seem counterintuitive to me, the data is data, and the findings are worth publishing. But it is strange collection of observations as if it was the beginning of several different stories, rather than one coherent story line. I see the beginnings of three stories: 1) the FRAP data on Figs 1 and 2, and the role of the ATPase mutation; 2) the role of chromatin modifications shown on Figs 3 and 4; and 3) the noncatalytic function of DPY-21 (Some data from Fig 4 and then Fig 5). Three stories, but none complete.

© 2021. Published by The Company of Biologists under the terms of the Creative Commons Attribution License (https://creativecommons.org/licenses/by/4.0/).

At the very least, I suggest changing some of the text (see details below).
Beyond that I do think that several observations would be worth pursuing much further and then publishing the stories when they are more complete and the findings are more impactful. But perhaps that is not the best strategy during covid.

Overall, the study reports some potentially interesting findings, but the mechanisms behind the observations are unclear. As I said above, after much more work, the data presented in this manuscript could potentially lead to three publications, each much more significant the current study. In its current form with some modifications, I still believe the data are publishable somewhere, I am just not sure about which journal might be most appropriate.

Comments for the author

The following suggestions all require changes to the text only:
1. The title does not accurately reflect the story. Yes, a few pieces of data from the paper talk about this noncatalytic ability of dpy-21, but only a few pieces, and we never figure out what this other activity is. Reading the title I expected to find out more about this additional activity, and less about the ATPase mutation and the chromatin modifiers.
2. In the introduction (top of P. 3) the authors present a model of DCC binding to the X chromosome by recruitment and spreading. Recent studies from the Meyer lab (Anderson et al 2019 Development Cell) and the Corces lab (Rowley et al 2020, Genome Research) revised this model somewhat. These papers propose that the complex binds the X chromosome at non-specific locations than it moves along the X by loop extrusion until it is blocked. I suggest that the authors include these models in their introduction.
3. Some of the experiments are incompletely described. For example, I was confused by the IPs on Figure 1D. The best I can tell, the IP was performed by the indicated antibodies, then the precipitate was western blotted with DPY-27 antibodies (according to the methods section at least). But then what are the extra bands? There should be two, one for the transgenic and one for the endogenous DPY-27. So then why are there different additional bands in each of the IPs?
4. Fig 1F, NLS::GFP is mispelled as NSL::GFP.
5. P. 14 middle paragraph, the authors state that “the mobile fraction free GFP (Figure 1G) and recovery half-life (Figure 1H) was much faster than that of H2B”. Since H2B was excluded from the Fig 1H, as the authors explain, this sentence should be revised.
6. I was not sure why Supplemental figure 1A was included. Since the GFP and Halo tags are never in the same strain, I wasn’t sure why it had to be demonstrated that the signals are separable. On the other hand, I thought the data on Supplemental Fig 1B was important enough to include in the main figure.
Maybe not all three replicates, but it is important to show that the GFP tagged DPY-27 binds to the X chromosomes correctly. As an aside, I found the cartoons with the complexes on the left side very helpful, so thank you for including them.
7. The section entitled “ATP hydrolysis is required…”. Technically, the authors did not show that the ATP hydrolysis ability was disrupted by the mutation they engineered. It is highly likely that it is, but it was not demonstrated. I would change the wording somewhat to clarify that a mutation that in other organisms disrupt ATP hydrolysis has this effect. I would also add that the conclusion (that the mutant protein does not bind) is only valid with the “in the presence of wild type protein” qualification, or something similar.
I would make this clear in the text. In fact the data on Fig 2 argues that DPY-26 preferentially interacts with the wild type proteins, and the mutant cannot compete it out.
8. The data on Supplemental Figure 2C shows that there is slight X chromosome derepression in the strain expressing the EQ mutant, but also in the strain expressing the wild type DPY-27 protein fused to GFP. The derepression is more significant for the mutant, but it is also measurable for the wild type.
The authors do not mention this in the text, and therefore also do not provide a (potential) explanation.
9. The data shown on Fig 3 is not very interesting, mostly negative data showing that DPY-27 binding does not change in chromatin modifier mutants. I was also unsure about why one would expect to see a change. My understanding is that these chromatin modifications are a consequence of condensin DC binding. Are the authors suggesting that once the complex binds, and these chromatin modifications appear, and they can then reinforce binding? (Except the data implies that they play no role.)

© 2021. Published by The Company of Biologists under the terms of the Creative Commons Attribution License (https://creativecommons.org/licenses/by/4.0/).

10. Along those lines, I would point out that the DPY-28 HEAT repeat binds to unmethylated H4K20 and monomethylated H4K20 peptide equally well (Figure 3D), so the complex could bind both before the modification is introduced, and also afterwards. Also, the HEAT repeat data does not lead to much conclusion. We can see some binding to nonacetylated H3 and H4 peptides, but binding is much reduced if the peptide is acetylated. However, the mutant data with sir-2.1 does not correlate with this, so it is either confusing, or the in vitro data is not relevant to in vivo binding.

11. The observation about the noncatalytic role of DPY-21 is perhaps the most interesting part of the story, but it is also confusing. FRAP data (Fig 3) indicates DPY-27 binds more tightly to the X in the dpy-21 null mutant. But the authors also cite previous ChIP-seq data that indicated somewhat reduced binding to the X in these mutants. I would like to see some discussion on how to reconcile these two pieces of information. I would also like to read some speculation about what this tighter binding might mean in terms of dosage compensation. X repression is lessened in the mutant—how is that a consequence of tighter binding of the complex? The authors suggest a role for DPY-21 as a condensin unloader, but I do not see how that can contribute to gene repression.

12. FRAP data Supp Fig 3: why is the FRAP recovery of background signal affected in the dpy-21 null?

13. I also found the Hi-C data (Fig 5) confusing. If dosage compensation is more disrupted in the dpy-21 null than in the dpy-21 JmjC mutant, how can the null rescue the Hi-C defects previously reported for the JmjC mutant? I would like to see the authors speculation on this question too. It just does not seem to make a lot of sense. I was almost wondering whether the differences were due to growth conditions or the ages of the embryos analyzed rather than true biologically significant consequences of the different mutations. Since the JmjC data was generated in a different lab, could this be an explanation?

Reviewer 3

Advance summary and potential significance to field

In this paper, the authors found that ATP hydrolysis by DPY-27 is required for localization of condensin DC to the X chromosome, and that non-catalytic part of DPY-21 regulates the dynamics of condensin DC. These findings will contribute to uncover how condensin DC is localized and released adequately. And study of condensin DC on the X chromosome will be a model for the localization study of condensin.

Comments for the author

These are comments to the regular paper entitled "A noncatalytic activity of the H4K20 demethylase DPY-21 regulates condensin DC binding".
In the paper, the authors studied how chromatin modifiers regulate condensin DC that is a X-chromosome-specific condensin in C. elegans. In former part, the authors constructed GFP-tagged DPY-27 variant strains and showed that ATP hydrolysis by DPY-27 is required for localization of condensin DC to the X chromosome by the FRAP experiment. In latter part, the author found that DPY-21 null mutant, but not DPY-21 catalytic mutant, reduced the proportion of mobile condensin DC, and suppressed the 3D DNA contacts on the X chromosome. Therefore, the authors insist that non-catalytic part of DPY-21 regulates the dynamics of condensin DC.

Major comments
The authors eagerly studied regulation of condensin DC on the X chromosome and found lots of new result in the manuscript. However, I suppose that the first half studying DPY-27 and the latter half studying DPY-21 are not functionally related in the manuscript. I feel there are two stories in the manuscript. The authors tried to connect the first half and the latter half by examining how condensin DC interacted with histone tails, but it was negative. I recommend separate the first half (Fig. 1 to 3) and the latter half (Fig. 4 to 6) or change logic of the manuscript when it is published. Because of same problem, the title does not represent the study of DPY-27, and summary in the beginning of discussion part does not include the study of DPY-21, on the contrary, summary in the last of discussion part does not include the study of DPY-27 .

© 2021. Published by The Company of Biologists under the terms of the Creative Commons Attribution License (https://creativecommons.org/licenses/by/4.0/).

Minor comments
In Figure 1D and Figure 2E, there is no lane for control (e.g. no-tag or flow-through) in the immuno-western blotting experiment, so that I am concerned about that the bands detected by anti-DPY-27 antibody are background. And, because immuno-western blotting membranes are separated, it is difficult to compare the position of several bands among the menbranes. The author should add any indicator of molecular weight beside three each membrane.
Page13 line24, the authors insisted that DPY-27 bound to DNA. However, these data showed just co-localization with DPY-27::Halo, condensin DC and X-chromosomes, but not direct 'binding'.
Page15 line23, the authors showed that DPY-27::GFP is more mobile that H2B::GFP. Is there any experimental data of the FRAP about other condensin proteins? Comparing the mobility among condensin proteins would support the special nature of DPY-27.
Page3 line8, 'Jumanji' is mistyping.
Page9 line10, 'thrice' is mistyping.
Page20 line18, co-immunoprecipitation experiment is in Figure 2E but not in 2F.

First revision

Author response to reviewers' comments

We thank the reviewers for their thoughtful assessment of our results and interpretations. Please see our response to each comment summarizing and highlighting the changes to the text, as well as the additional experiments and explanations. Throughout the text below, the original reviewer comments are provided in black and our response is in blue color.

Reviewer 1

Main points

Fig 2E: The ratio of endogenous DPY-27 to DPY-27::GFP signal should be calculated and graphed in both WT and EQ contexts to support the authors' assertion that EQ is less likely to interact with DPY-26 than WT. The images show proportional decreases of both endogenous and tagged DPY-27 at both time points in WT and EQ strains.

To further increase confidence that DPY-26 IPed wt-DPY-27 better than EQ-DPY-27, we added data in embryos (Supplemental Figure 2F). Multiple bands in the adults prevent us from making quantitative measurements on the ratio of GFP-endogenous (Supplemental Figure 1B). We are confident that the antibody is specific (Kramer et al. 2015 Plos Gen. Supplemental Figure 4B), but we noticed the appearance of multiple bands in young adults in some blots and not others, therefore we are not sure whether the bands are background or degradation products. Based on the embryo data, which agrees with the results from the young adults, we conclude that the WT-DPY-27 interacts better with DPY-26 compared to EQ-DPY-27.

Furthermore, the authors claims regarding proteins stability (discussion of Fig 2B in text) seems out of sync with the data. "Directly after heat shock… expressed at similar levels" the figure does not show signal intensity directly after heat shock (what time point does 'directly' indicate?). Add pixel intensity of this 'direct' time point to the graphs. By 8 hr, both WT and EQ appear similar in their signal, also undermining claims of EQ-specific protein degradation. The authors should provide median signal intensity for the 'direct after heat shock', 3hr and 8 hr time point to clarify these claims. As currently shown, there is less overall decrease of signal in the EQ mutant going from 3 hr to 8 hr (WT (3x less, EQ 2x less). The relevant section of the discussion should be modified accordingly.

Upon the reviewer's comment, we quantified the level of reduction and added additional example images. Indeed, we were unable to find a statistically significant difference between the reduction of total signal between the WT and EQ. We present the additional data in Supplemental Figures 2D and 2E and remove any comments and conclusions on stability from the manuscript.

© 2021. Published by The Company of Biologists under the terms of the Creative Commons Attribution License (https://creativecommons.org/licenses/by/4.0/).

Also, the methods suggest that separate mosSCI transgenes, one WT and one EQ, were separately integrated. Independent mosSCI integration events, even of the same transgenes, can show quite variable expression. Therefore, any discussion of protein levels needs to explicitly acknowledge this caveat to interpreting total protein levels.

We recognize that the total protein level may be different between the WT and the EQ proteins. We also noted in the manuscript that heat shock produces different protein expressions. Our conclusion that EQ-DPY-27 is not binding to the X chromosomes is robust. We show that EQ protein is being expressed in Figure 2B, Figure 2E, Supplemental Figure 2D-F.

HEAT in vitro binding of recombinant histone peptides: The figures show data consistent with worm dosage compensation. However, either the text or the representative image does not clearly describe the K20 methylation data. Specifically, there appears to be no difference in K20me0 and K20me1, with less binding to me2/3. Yet, the text says ‘increased methylation of lysine 20 [relative to unmodified, from the previous sentence] reduced the interaction. The authors need to quantify the binding intensities, normalized to peptide loading, and display the ratios me1 vs me0, me2 vs me0, me3 vs me0, etc. They need to perform statistical analysis of the difference in signal and indicate in the legend how many independent replicates were analyzed. Finally, the text would need to be appropriately modified. Since DC is associated with K20me1 enrichment at the expense of K20me2/3, this difference matters.

Unfortunately, in solution peptide binding experiments using western blot analysis is not quantitative enough to provide robust quantitative comparisons of binding, thus we refrain from overinterpretation, especially because we did not see any effect of modification changes on condensin DC binding measured by ChIP-seq. The replicates support that the HEAT domain binds to the histone peptides, and interaction is reduced slightly by K20me3 and to a greater extent by H4-tetraacetyl. We revised the sentence as “Tetra-acetylation and trimethylation of lysine 20 reduced the interaction (Fig. 3D)“.

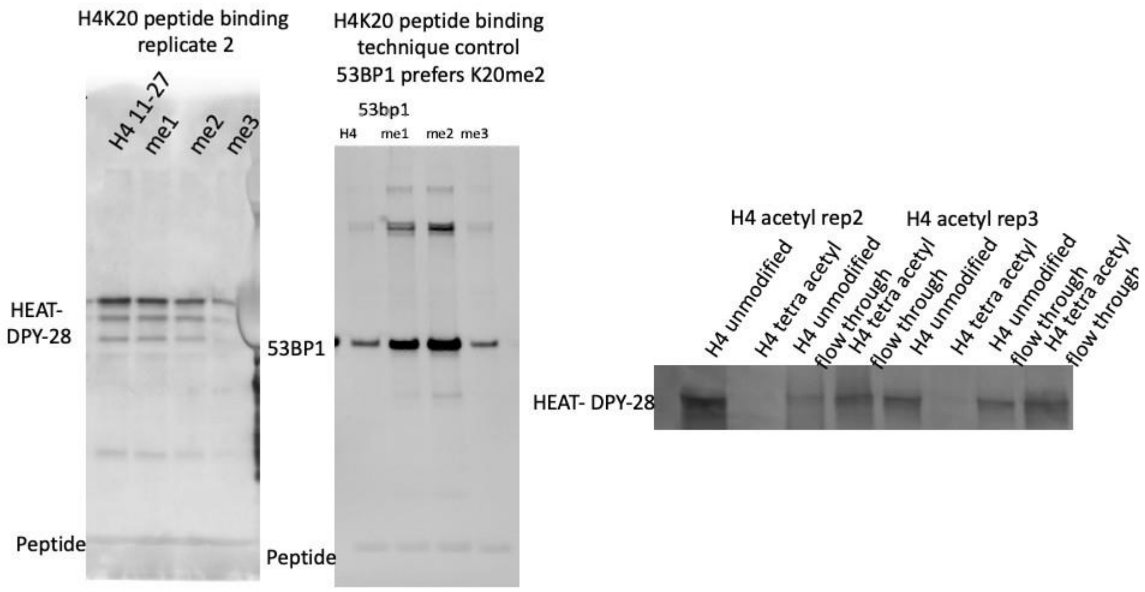

The RNAi feeding strategy is missing from the methods. This should be added. Specifically, it is problematic for interpreting set-1 RNAi experiments (Fig 4A/B). If knockdown of set-1 is induced after embryogenesis, it is possible that its role in condensin DC motility may have already been performed. Therefore, set-1 RNAi feeding should be started no later than the late larval stage L4 in the generation prior to the one in which animals are imaged. The authors are requested to clarify the method used and, if necessary, address the timing of RNAi addition with new experiments. SET-4 has been implicated in DC in several studies.

We added that the FRAP experiment for *set-1* RNAi is performed in young adults by starting feeding at the L1 stage (methods section). Set-1 knock-down results in germline defects, therefore FRAP

© 2021. Published by The Company of Biologists under the terms of the Creative Commons Attribution License (https://creativecommons.org/licenses/by/4.0/).

was performed in worms lacking a germline. SDC-2 recruits the complex to the X chromosome, and as expected, we observe a lack of DPY-27 localization (Figure 2D) using our RNAi feeding strategy. Additionally, we monitored the efficiency of our RNAi knock-down by including a positive control with the embryonic lethal knock-down for pop-1, which results in dead embryos which were compared to empty vector treatment.
It is difficult to interpret the negative data of why set-1 RNAi does not affect DPY-27 FRAP. We refrain from making any conclusions and simply state that there is no effect on binding given the experimental setup.

Here, the authors show that loss of set-4 increases dumpiness in animals. In Fig 4A set-4 also seems to decrease FRAP recovery. However, the authors ignore this difference. Please clarify either with statistical measurements or supporting information that justifies this interpretation of the data. Furthermore, the authors' claim that the H4K16/K20 modifiers act downstream of condensin DC is incompletely explored. Specifically, perhaps H4K20me2 traps condensin DC on chromatin in the absence of DPY-21 protein, and the non-catalytic activity senses or reduces interaction with this PTM in some way. The authors are encouraged to perform FRAP recovery in animals null for both dpy-21 and set-4 alleles to address this question.

Thank you for highlighting *set-4*, we added that there is a statistically significant difference from the wild type. The modifiers downstream of DPY-21 are indeed underexplored, and future work should address this possibility.

Missing is some comment regarding the nature of the non-catalytic activity. Is there structural analysis possible that would provide a testable basis for a mechanism (e.g. the K20me2 experiment proposed above)?

We agree that the next step in the project would be to use structural analysis to understand the nature of the non-catalytic activity, especially as part of the model the reviewer presented in DPY-21 regulating the possible interaction of condensin with histones. Lack of any structural work on the DPY-21 protein itself (outside the JmjC domain) does not allow for stronger speculations. But we did add a possible scaffolding function and DPY-21 possibly regulating condensin DC interaction with histone tails in the discussion.

Finally, the manuscript would benefit from performing FRAP experiments also in early embryos because comparisons could be made between condensin DC dynamics in dividing and non-dividing cells. Although HS induction may be variable, the images in Fig S1C suggest the localization to X is discernible, and C. elegans embryonic subnuclear concentration of proteins have been successfully FRAP'ed before. Imaging embryos between the 50-100 cell stage would likely yield nuclei sufficiently large to FRAP at a developmental stage in which DC has been activated.

This is an excellent point and we had hoped to do the analysis in embryos, however, the nuclei are very small and we are not able to use our current setup. More high-resolution analysis of the complex binding in the future can address the difference between dividing and non-dividing cells. Previous results performing FRAP in embryos often used very early embryos or cellular membranes but not subnuclear structures in older embryos (which have smaller cells) after DCC binding.

Minor points

Even though the endogenously tagged GFP allele was not suitable for analysis, please include the details of insert preparation in the methods as done for the halo tag. NOTE: "Generation of Halo::DPY-27 strain" should be written DPY-27::Halo if, as the method says, Halo was attached to the C-terminus.

We corrected the text in the method section to read DPY-27::Halo.

Fig 1B: indicate the post-heat shock time point.

We indicated the post-heat shock time point in the revised figure legend.

Fig 1C: show representative images for 0/3/8 hr.

© 2021. Published by The Company of Biologists under the terms of the Creative Commons Attribution License (https://creativecommons.org/licenses/by/4.0/).

We show the images in the revised figure.

Fig 1D: indicate visually and in the legend that anti-DPY-27 antibody was used for blotting.

We corrected the text accordingly.

Fig 1G: indicate the time point at which the mobility fraction is calculated

The mobile fraction is calculated at the last recorded time point of every single experiment after the blech curve reached a plateau. Since this timepoint slightly varies between each experiment, it is difficult to mark it directly in the FRAP recovery curve. We added however this sentence to the methods to make this more clear: “The mobile fraction was calculated from the monoexponential fit at the last recorded recovery time point of each experiment.”

“To test if the failure of DPY-27(EQ)::GFP to bind is due to its inability to form a complex, we performed co-immunoprecipitation experiments (Figure 2F).” Should be 2E.

We corrected the text accordingly.

Fig S2C Add X:A RNAseq data from a DC mutant to this graph to provide context. Using the dpy-21 (Kramer 2015) data again would suffice. Please also address in the text that there is X:A imbalance in the WT DPY-27::GFPstrain.

We added mRNA-seq data for *dpy-21 (e428)* by Kramer et al. 2015 to figure S2C to provide better context. We also added a sentence about X-upregulation in WT DPY-27::GFP strain.

“Thus, set-1, set-4, sir-2.1, and the catalytic activity of dpy-21 act downstream of condensin DC…” binding? Dynamics? Clarify which aspect of condensin these genes act downstream of, since they participate in X chromosome silencing too. The same comment is relevant to the discussion: “Thus, H4K20me1 and H4K16ac act downstream of condensin DC [what - binding or dynamics ?] to repress transcription.”

Yes, we did mean downstream of condensin DC binding since we did not notice a change in ChIP-seq and small changes in FRAP. We edited the sentences accordingly.

Same problem in first two discussion paragraphs: “also eliminates DPY-27 binding (to what) (Fig 2)” and DPY-27 EQ mutation eliminating its binding (to what), measured by…”

These are edited to read “.... binding to the X chromosomes “

Fig S3E WT tracks missing from figure but indicated as present in figure legend.

We fixed the legend text for figure S3E.

17 nuclei (Fig 4C) are insufficient to 1) get such a smooth a distribution and 2) to be robustly compared with 31 dpy-21 nuclei, to give a tail of high signal. The difference should be tested with statistics and more control nuclei should be scored..

We added additional replicates to increase the total to 27 nuclei for the wild-type condition and 35 nuclei for the *dpy-21* KO condition for figure 4C. Below, we show data as a histogram in addition to smooth density plots. In the *dpy-21* KO, there are more high-intensity pixels. A Mann-Whitney U test confirms that the distributions of pixel intensities are significantly different between the two conditions with a p-value of $1.46 * 10^{-114}$.

© 2021. Published by The Company of Biologists under the terms of the Creative Commons Attribution License (https://creativecommons.org/licenses/by/4.0/).

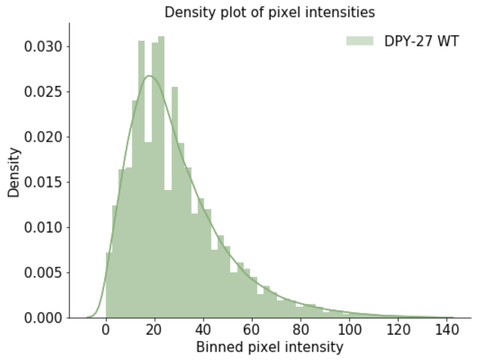

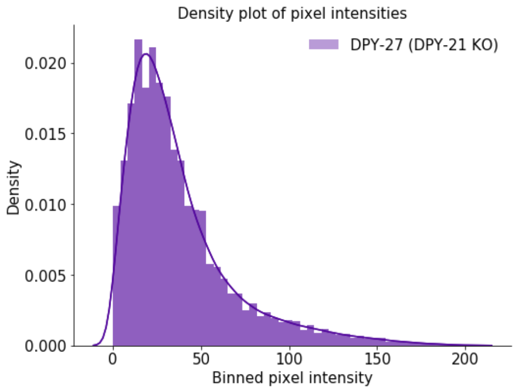

Reviewer 2

1. The title does not accurately reflect the story. Yes, a few pieces of data from the paper talk about this noncatalytic ability of dpy-21, but only a few pieces, and we never figure out what this other activity is. Reading the title I expected to find out more about this additional activity, and less about the ATPase mutation and the chromatin modifiers.

We changed the title to "The H4K20 demethylase DPY-21 regulates the dynamics of condensin DC binding"

2. In the introduction (top of P. 3) the authors present a model of DCC binding to the X chromosome by recruitment and spreading. Recent studies from the Meyer lab (Anderson et al 2019 Development Cell) and the Corces lab (Rowley et al 2020, Genome Research) revised this model somewhat. These papers propose that the complex binds the X chromosome at non-specific locations than it moves along the X by loop extrusion until it is blocked. I suggest that the authors include these models in their introduction.

Anderson et al. propose a model where the complex binds to non-specific locations and moves along the X until hitting the rex sites (recruitment elements on the X). We believe this model is appropriate for a steady-state/maintenance stage, once X chromosome-wide binding is established, but cannot explain the initial and specific recruitment of condensin DC to the X chromosome. We edited the introduction paragraph to add the steady-state models.

Below we also elaborate on why recruitment remains the likely model for X-specific localization of condensin DC. Initial extrachromosomal array experiments that identified the rex elements show that they recruit condensin DC. These experiments showed that only those arrays containing rex sequences are bound by condensin DC (McDonel et al 2006 PMID:17122774, Jans et al 2009 PMID: 19270160). In the absence of rex sequences, there is no DPY-27 immunofluorescence signal on the arrays. Importantly, multi-copy rex sequences deplete condensin DC from the X (see panel f figure 2 McDonel et al 2006 PMID:17122774). This definitively demonstrates that the elements "recruit the complex".

The point of argument is whether the recruitment elements act as condensin DC "loading sites" or "stick" to the complex to "retain" condensin DC on the X. We do not elaborate on these distinctions because it remains unknown how SDC-2 protein controls condensin DC localization to the X chromosomes. Anderson et al also recognize that "loading" and "blocking" models are not exclusive of each other, quote: *"Thus, SDC-2 could be responsible both for loading the DCC onto X at all rex sites and rex-dependent secondary sites (as NIPBL loads cohesin) and for binding the highest-affinity rex sites in a manner that blocks loop extrusion (as CTCF apparently blocks cohesin extrusion). In principle, SDC-2 could travel with DCC condensin from loading sites on X to the highest-affinity rex sites where SDC-2 binds most stably and blocks extrusion." "Alternatively, because SDC-2 can bind rex sites independently of condensin, and condensin can bind secondary sites on X at a low level in the absence of SDC-2 (Albritton et al., 2017; Pferdehirt et al., 2011), condensin alone could extrude a loop until encountering SDC-2 bound at a rex site." "A*

© 2021. Published by The Company of Biologists under the terms of the Creative Commons Attribution License (https://creativecommons.org/licenses/by/4.0/).

combination of both options might occur."

3. Some of the experiments are incompletely described. For example, I was confused by the IPs on Figure 1D. The best I can tell, the IP was performed by the indicated antibodies, then the precipitate was western blotted with DPY-27 antibodies (according to the methods section at least). But then what are the extra bands? There should be two, one for the transgenic and one for the endogenous DPY-27. So then why are there different additional bands in each of the IPs?

We clarified the IP/hybridization in the figure legend. For multiple bands seen in the young adult western blots, please see our comment to reviewer 1 (second point).

4. Fig 1F, NLS::GFP is mispelled as NSL::GFP.

This is corrected.

5. P. 14 middle paragraph, the authors state that "the mobile fraction free GFP (Figure 1G) and recovery half-life (Figure 1H) was much faster than that of H2B". Since H2B was excluded from the Fig 1H, as the authors explain, this sentence should be revised.

"Recovery half-life (Figure 1H)" is removed from the sentence

6. I was not sure why Supplemental figure 1A was included. Since the GFP and Halo tags are never in the same strain, I wasn't sure why it had to be demonstrated that the signals are separable. On the other hand, I thought the data on Supplemental Fig 1B was important enough to include in the main figure. Maybe not all three replicates, but it is important to show that the GFP tagged DPY-27 binds to the X chromosomes correctly. As an aside, I found the cartoons with the complexes on the left side very helpful, so thank you for including them.

In supplemental figure 1 A DPY-27 GFP and Halo are indeed in the same strain and co-localize, thus demonstrating that DPY-27 GFP protein localizes the same sites as the endogenously Halo tagged protein.

We show average ChIP binding for wild-type DPY-27 in Figure 2C, where the comparison to EQ is important. To prevent repetition, we decided not to include the ChIP data in the main Figure 1 but referred to Supplemental Figure 1B. We also referred to Figure 2C in the revised text.

7. The section entitled "ATP hydrolysis is required…". Technically, the authors did not show that the ATP hydrolysis ability was disrupted by the mutation they engineered. It is highly likely that it is, but it was not demonstrated. I would change the wording somewhat to clarify that a mutation that in other organisms disrupt ATP hydrolysis has this effect. I would also add that the conclusion (that the mutant protein does not bind) is only valid with the "in the presence of wild type protein" qualification, or something similar.
I would make this clear in the text. In fact the data on Fig 2 argues that DPY- 26 preferentially interacts with the wild type proteins, and the mutant cannot compete it out.

We agree and edited the subheading, which now reads as "A conserved mutation to the DPY-27 ATPase domain eliminates its binding in the presence of the wild type protein". We also added the following sentence to the discussion "...and although the EQ mutation reduced ATP hydrolysis in all SMC complexes analyzed so far, future work is needed to characterize the specific effect of this mutation on condensin DC."

8. The data on Supplemental Figure 2C shows that there is slight X chromosome derepression in the strain expressing the EQ mutant, but also in the strain expressing the wild type DPY-27 protein fused to GFP. The derepression is more significant for the mutant, but it is also measurable for the wild type.
The authors do not mention this in the text, and therefore also do not provide a (potential) explanation.

In the results, we added a sentence "X upregulation upon wild type DPY-27::GFP expression may be due to dosage imbalance within the complex. "

© 2021. Published by The Company of Biologists under the terms of the Creative Commons Attribution License (https://creativecommons.org/licenses/by/4.0/).

9. The data shown on Fig 3 is not very interesting, mostly negative data showing that DPY-27 binding does not change in chromatin modifier mutants. I was also unsure about why one would expect to see a change. My understanding is that these chromatin modifications are a consequence of condensin DC binding. Are the authors suggesting that once the complex binds, and these chromatin modifications appear, and they can then reinforce binding? (Except the data implies that they play no role.)

Yes, we hypothesized that there might be feedback given that condensin has the potential to bind to histones (HEAT domain binding to histone tails), but the prediction of this hypothesis was not met. We present it as negative data and conclude as such.

10. Along those lines, I would point out that the DPY-28 HEAT repeat binds to unmethylated H4K20 and monomethylated H4K20 peptide equally well (Figure 3D), so the complex could bind both before the modification is introduced, and also afterwards. Also, the HEAT repeat data does not lead to much conclusion. We can see some binding to nonacetylated H3 and H4 peptides, but binding is much reduced if the peptide is acetylated. However, the mutant data with sir-2.1 does not correlate with this, so it is either confusing, or the in vitro data is not relevant to in vivo binding.

HEAT repeat binding data raised the possibility that perhaps histone modifications affect condensin DC binding. ChIP-seq showed that modifier mutants don't have an effect. Note that the mutants reduce or increase the modifications on the X. For example in sir-2.1 null, the level of H4K16 acetylation is increased on X chromosomes by 2-fold. So the effect could be subtle and more sensitive assays may capture it in the future.

11. The observation about the noncatalytic role of DPY-21 is perhaps the most interesting part of the story, but it is also confusing. FRAP data (Fig 3) indicates DPY-27 binds more tightly to the X in the dpy-21 null mutant. But the authors also cite previous ChIP-seq data that indicated somewhat reduced binding to the X in these mutants. I would like to see some discussion on how to reconcile these two pieces of information. I would also like to read some speculation about what this tighter binding might mean in terms of dosage compensation. X repression is lessened in the mutant—how is that a consequence of tighter binding of the complex? The authors suggest a role for DPY-21 as a condensin unloader, but I do not see how that can contribute to gene repression.

We added the following paragraph to the discussion:
"How do the catalytic and noncatalytic activities of DPY-21 contribute to repression? DPY-21 mediated enrichment of H4K20me1 leads to reduction of H4K16ac on the X chromosomes, which may reduce binding of general activator(s), contributing a portion of the observed 2-fold repression provided by condensin DC (Sheikh et al., 2019). Our work suggests that a non-catalytic activity of DPY-21 contributes to repression by regulating the kinetics of condensin DC diffusion. In the *dpy-21* null mutant, but not in the *JmjC* mutant, the fraction of mobile condensin DC reduced from ~30% to ~10%. Interestingly, in the *dpy-21* null mutant, condensin DC binding to promoters slightly decreases (Kramer et al., 2015), and the DPY-27::Halo signal shows higher intensity spots. It is possible that, without DPY-21, condensin DC is more frequently "trapped" in an immobile configuration that reduces condensin DC presence and activity at promoters that represses transcription initiation. "

12. FRAP data Supp Fig 3: why is the FRAP recovery of background signal affected in the dpy-21 null?

DPY-21 is known to have functions outside of dosage compensation (see discussion) thus it may also be affecting autosomal chromatin or gene regulation.

13. I also found the Hi-C data (Fig 5) confusing. If dosage compensation is more disrupted in the dpy-21 null than in the dpy-21 JmjC mutant, how can the null rescue the Hi-C defects previously reported for the JmjC mutant? I would like to see the authors speculation on this question too. It just does not seem to make a lot of sense. I was almost wondering whether the differences were due to growth conditions or the ages of the embryos analyzed rather than true biologically significant consequences of the different mutations. Since the JmjC data was generated in a different lab, could this be an explanation?

© 2021. Published by The Company of Biologists under the terms of the Creative Commons Attribution License (https://creativecommons.org/licenses/by/4.0/).

We took the possibility of using data from a different lab seriously and decided to invest in this question. We repeated the Hi-C in JmjC mutant embryos. We found a slight reduction in short-range interactions but did not observe a loss of *rex-rex* loops in the JmjC mutant as reported in Brejc et al. 2017. Although it is not clear what the cause of the discrepancy is (we obtained the mutant from Meyer lab and validated the genotype using Hi-C reads), this new result simplified our interpretation and made interpretation less confusing, as explained in the updated discussion.

Reviewer 3

Major comments

The authors eagerly studied regulation of condensin DC on the X chromosome and found lots of new result in the manuscript. However, I suppose that the first half studying DPY-27 and the latter half studying DPY-21 are not functionally related in the manuscript. I feel there are two stories in the manuscript. The authors tried to connect the first half and the latter half by examining how condensin DC interacted with histone tails, but it was negative. I recommend separate the first half (Fig. 1 to 3) and the latter half (Fig. 4 to 6) or change logic of the manuscript when it is published. Because of same problem, the title does not represent the study of DPY-27, and summary in the beginning of discussion part does not include the study of DPY-21, on the contrary, summary in the last of discussion part does not include the study of DPY-27 .

We think that having the FRAP and the ChIP/Hi-C portions of the manuscript is important because the striking difference between the DPY-21 null and catalytic mutant is measured by FRAP but not ChIP or Hi-C. While it was difficult to reconcile these observations, we hope the additions to the discussion helps to connect the observations. We changed the title to be more descriptive of the work.

Minor comments

In Figure 1D and Figure 2E, there is no lane for control (e.g. no-tag or flow-through) in the immuno-western blotting experiment, so that I am concerned about that the bands detected by anti-DPY-27 antibody are background. And, because immuno-western blotting membranes are separated, it is difficult to compare the position of several bands among the menbranes. The author should add any indicator of molecular weight beside three each membrane.

We added the molecular weights and showed additional western blots including non heat shock and heat shock samples. For more information, please see our comments to Reviewer 1 point 2.

Page13 line24, the authors insisted that DPY-27 bound to DNA. However, these data showed just co-localization with DPY-27::Halo, condensin DC and X-chromosomes, but not direct ‘binding’.

The conclusion is supported by multiple lines of evidence including our WT DPY-27-GFP ChIP-seq (Figure 2C) and previous studies showing that without binding, DPY-27 is diffusely distributed within the nucleus and does not show X-specific localization and is unable to perform dosage compensation (PMID: 11937488, PMID: 8939869, PMID: 7954812). Also in agreement with the literature, knockdown of SDC-2 eliminated X-specific localization of WT DPY-27 (Figure 2D). While there may be different modes of “binding” to DNA, we tried to clarify that we refer to X chromosomal localization throughout the manuscript.

Page15 line23, the authors showed that DPY-27::GFP is more mobile that H2B::GFP. Is there any experimental data of the FRAP about other condensin proteins? Comparing the mobility among condensin proteins would support the special nature of DPY-27.

We have compared our results to condensins from other organisms but not in *C. elegans*. Therefore it remains unclear if DPY-27 and SMC-4 differ, and could be a question to be answered in future work.

Page3 line8, ‘Jumanji’ is mistyping.

We corrected the text accordingly.

© 2021. Published by The Company of Biologists under the terms of the Creative Commons Attribution License (https://creativecommons.org/licenses/by/4.0/).

Page9 line10, ‘thrice’ is mistyping.

We are not clear about this correction.

Page20 line18, co-immunoprecipitation experiment is in Figure 2E but not in 2F.

We corrected the text accordingly.

Second decision letter

MS ID#: JOCES/2021/258818

MS TITLE: The H4K20 demethylase DPY-21 regulates the dynamics of condensin DC binding.

AUTHORS: Laura Breimann, Ana Karina Morao, Jun Kim, David Jimenez, Nina M. Maryn, Krishna Bikkasani, Michael J Carrozza, Kustrim Cerimi, Vic-Fabienne Schumann, Sarah Elizabeth Albritton, Maxwell Kramer, Lena Annika Street, Ella Bahry, Stephan Preibisch, Andrew Woehler, and Sevinc Ercan
ARTICLE TYPE: Research Article

We have now reached a decision on the above manuscript.

To see the reviewers' reports and a copy of this decision letter, please go to: https://submit-jcs.biologists.org and click on the 'Manuscripts with Decisions' queue in the Author Area. (Corresponding author only has access to reviews.)

As you will see, the reviewers gave favourable reports but raised some critical points that will require amendments to your manuscript. I hope that you will be able to carry these out because I would like to be able to accept your paper. In particular, please do the textual changes that Reviewer 1 points out, and please provide an explanation for the differences between your Hi-C data and that published by Brejc et al, as requested by Reviewer 2.

We are aware that you may be experiencing disruption to the normal running of your lab that makes experimental revisions challenging. If it would be helpful, we encourage you to contact us to discuss your revision in greater detail. Please send us a point-by-point response indicating where you are able to address concerns raised (either experimentally or by changes to the text) and where you will not be able to do so within the normal timeframe of a revision. We will then provide further guidance. Please also note that we are happy to extend revision timeframes as necessary.

Please ensure that you clearly highlight all changes made in the revised manuscript. Please avoid using 'Tracked changes' in Word files as these are lost in PDF conversion.

I should be grateful if you would also provide a point-by-point response detailing how you have dealt with the points raised by the reviewers in the 'Response to Reviewers' box. Please attend to all of the reviewers' comments. If you do not agree with any of their criticisms or suggestions please explain clearly why this is so.

Reviewer 1

Advance summary and potential significance to field

This report provides a valuable survey of different attributes of the condensin DC complex that govern its behavior (ATP hydrolysis, domains of interaction with chromatin, dynamic association with chromatin), as well as its relationship with chromatin PTMs. Despite the fact that some data are interpreted purely on qualitative terms (some but not all being quantified now) -

© 2021. Published by The Company of Biologists under the terms of the Creative Commons Attribution License (https://creativecommons.org/licenses/by/4.0/).

the paper is valuable and is important to the field.

Comments for the author

The revised paper addresses most of the points raised. There are some minor issues to correct in the typing. Additional minor changes requested are below:
Fig 4C legend. Please indicate that the data is smoothed and include the unsmoothed histograms presented in the response to reviewers in the supplement with the same x-axis scale for both WT and dpy-21 graphs.

Text: “Second, DPY-27::GFP immunoprecipated…”. The logic of this sentence is backwards. Rather, it should read something like: “Second, DPY-27::GFP was detected in immunoprecipitation of DC subunits, supporting complex formation.”

Typo: Intro first paragraph: “where an X-specific condensing binding and function..” remove ‘an’

<u>Reviewer 2</u>

Advance summary and potential significance to field

My original main concern about the manuscript was that it did not seem to be one coherent study. Although the quality of some of the data improved in the revision, my main concern remains. Reviewer 3 seemed to agree with my assessment. Furthermore, much of the data is negative. And as the authors admit it’s hard to make firm conclusions based on negative data. However, when the authors “refrain from making any conclusions and simply state that there is no effect on binding given the experimental setup”, the readers do not learn much from the experiment. Whether JCS is willing to publish a paper with the beginnings of several stories as one study, with a significant portion of the data being negative, I suppose is an editorial decision. There is a lot of useful new data in this manuscript. But the story as presented seems a bit disjointed.

The newly added data, specifically the new Hi-C data in the dpy-21 catalytic mutants raised an additional concern. The observation that the catalytic mutant looks similar to the null mutant makes the interpretation of the data presented in this study less confusing. However, it is hard to reconcile it with previously published data (Brejc et al 2017) that used the same C. elegans strain and the same technique and came to a different conclusion. It worries me that two labs that are proficient in this type of analysis are able to come to opposite conclusions. Especially given that the original study saw a difference between mutant and wild type, but the current study was unable to detect differences. I would like to see an explanation. Are their differences in the protocols that could account for the difference? A difference in developmental stage (both studies used embryos, but maybe in one study the embryos were slightly older)? A difference in the analysis pipeline?

Comments for the author

There should be an explanation for the differences between the Hi-C data in this study and the paper by Brejc et al.
Otherwise, if the authors wish to publish this set of data as one story, I think they did as good a job as possible to try to connect the different pieces together.

© 2021. Published by The Company of Biologists under the terms of the Creative Commons Attribution License (https://creativecommons.org/licenses/by/4.0/).

Reviewer 3

Advance summary and potential significance to field

-

Comments for the author

The authors revised the manuscript well. DPY-21 may interact with condensin DC through binding to histone tail and control the mobility of condensin DC in C. elegans, which is a future work. I accept the revised manuscript and recommed it to be published on the JCS.

Second revision

Author response to reviewers' comments

Reviewer 1 Advance Summary and Potential Significance to Field:
This report provides a valuable survey of different attributes of the condensin DC complex that govern its behavior (ATP hydrolysis, domains of interaction with chromatin, dynamic association with chromatin), as well as its relationship with chromatin PTMs. Despite the fact that some data are interpreted purely on qualitative terms (some but not all being quantified now) - the paper is valuable and is important to the field.

Reviewer 1 Comments for the Author:
The revised paper addresses most of the points raised. There are some minor issues to correct in the typing. Additional minor changes requested are below:

Fig 4C legend. Please indicate that the data is smoothed and include the unsmoothed histograms presented in the response to reviewers in the supplement with the same x-axis scale for both WT and dpy-21 graphs.

We added the information of the smoothing to the figure legend and included the separate histograms to the supplements (S4C).

Text: "Second, DPY-27::GFP immunoprecipitated…". The logic of this sentence is backwards. Rather, it should read something like: "Second, DPY-27::GFP was detected in immunoprecipitation of DC subunits, supporting complex formation."

Thank you for highlighting this mistake. The logic of the sentence was wrong and we corrected it to read: "Second, DPY-27::GFP was detected in immunoprecipitation of DC subunits, supporting the complex formation capabilities of DPY-27::GFP"

Typo: Intro first paragraph: "where an X-specific condensing binding and function.."
remove 'an'

We removed "an" from this sentence.

Reviewer 2 Advance Summary and Potential Significance to Field:
My original main concern about the manuscript was that it did not seem to be one coherent study. Although the quality of some of the data improved in the revision, my main concern remains. Reviewer 3 seemed to agree with my assessment. Furthermore, much of the data is negative. And as the authors admit, it's hard to make firm conclusions based on negative data. However, when the authors "refrain from making any conclusions and simply state that there is no effect on binding given the experimental setup", the readers do not learn much from the experiment. Whether JCS is willing to publish a paper with the beginnings of several stories as one study, with a significant portion of the data being negative, I suppose is an editorial decision. There is a lot of useful new data in this manuscript. But the story as presented seems a bit disjointed.

© 2021. Published by The Company of Biologists under the terms of the Creative Commons Attribution License (https://creativecommons.org/licenses/by/4.0/).

The newly added data, specifically the new Hi-C data in the dpy-21 catalytic mutants raised an additional concern. The observation that the catalytic mutant looks similar to the null mutant makes the interpretation of the data presented in this study less confusing. However, it is hard to reconcile it with previously published data (Brejc et al 2017) that used the same C. elegans strain and the same technique and came to a different conclusion. It worries me that two labs that are proficient in this type of analysis are able to come to opposite conclusions. Especially given that the original study saw a difference between mutant and wild type, but the current study was unable to detect differences. I would like to see an explanation. Are their differences in the protocols that could account for the difference? A difference in developmental stage (both studies used embryos, but maybe in one study the embryos were slightly older)? A difference in the analysis pipeline?

Reviewer 2 Comments for the Author:
There should be an explanation for the differences between the Hi-C data in this study and the paper by Brejc et al. Otherwise, if the authors wish to publish this set of data as one story, I think they did as good a job as possible to try to connect the different pieces together.

We added a section in results addressing the differences between our Hi-C and published Hi-C data in wild type and *dpy-21(jmjC)* mutant (page 10). We provided several plots for each replicate from both studies, all analyzed through our pipeline. This shows that the difference is not due to the data analysis pipeline. While difference in the stage of embryos is one possibility, there is no way to test it using the Hi-C data. Another difference may be the way embryos were crosslinked. Crosslinking was shown to affect Hi-C data, and indeed Brejc et al use a lighter crosslinking regime compared to us. In the revised version of our manuscript, we provide all the plots as Supplemental Figures 6 and 7 and discuss how crosslinking could affect and lead to the differences in the results section.

Reviewer 3 Advance Summary and Potential Significance to Field:

Reviewer 3 Comments for the Author:
The authors revised the manuscript well. DPY-21 may interact with condensin DC through binding to histone tail and control the mobility of condensin DC in C. elegans, which is a future work. I accept the revised manuscript and recommed it to be published on the JCS.

Thank you for your input. We did edit the model figure to highlight this possibility in the final version.

Third decision letter

MS ID#: JOCES/2021/258818

MS TITLE: The H4K20 demethylase DPY-21 regulates the dynamics of condensin DC binding.

AUTHORS: Laura Breimann, Ana Karina Morao, Jun Kim, David Jimenez, Nina M. Maryn, Krishna Bikkasani, Michael J Carrozza, Kustrim Cerimi, Vic-Fabienne Schumann, Sarah Elizabeth Albritton, Maxwell Kramer, Lena Annika Street, Ella Bahry, Stephan Preibisch, Andrew Woehler, and Sevinc Ercan
ARTICLE TYPE: Research Article

I am happy to tell you that your manuscript has been accepted for publication in Journal of Cell Science, pending standard ethics checks.

© 2021. Published by The Company of Biologists under the terms of the Creative Commons Attribution License (https://creativecommons.org/licenses/by/4.0/).
